# Population structure and genetic basis of the agronomic traits of upland cotton in China revealed by a genome‐wide association study using high‐density SNPs

**DOI:** 10.1111/pbi.12722

**Published:** 2017-04-12

**Authors:** Cong Huang, Xinhui Nie, Chao Shen, Chunyuan You, Wu Li, Wenxia Zhao, Xianlong Zhang, Zhongxu Lin

**Affiliations:** ^1^ National Key Laboratory of Crop Genetic Improvement College of Plant Sciences & Technology Huazhong Agricultural University Wuhan Hubei China; ^2^ Key Laboratory of Oasis Ecology Agricultural of Xinjiang Bingtuan Agricultural College Shihezi University Shihezi Xinjiang China; ^3^ Cotton Research Institute Shihezi Academy of Agriculture Science Shihezi Xinjiang China; ^4^ Economic Crop Research Institute Henan Academy of Agricultural Sciences Zhengzhou Henan China

**Keywords:** cotton, yield, fibre quality, single‐nucleotide polymorphism, population structure, genome‐wide association study

## Abstract

*Gossypium hirsutum* L. represents the largest source of textile fibre, and China is one of the largest cotton‐producing and cotton‐consuming countries in the world. To investigate the genetic architecture of the agronomic traits of upland cotton in China, a diverse and nationwide population containing 503 *G. hirsutum* accessions was collected for a genome‐wide association study (GWAS) on 16 agronomic traits. The accessions were planted in four places from 2012 to 2013 for phenotyping. The CottonSNP63K array and a published high‐density map based on this array were used for genotyping. The 503 *G. hirsutum* accessions were divided into three subpopulations based on 11 975 quantified polymorphic single‐nucleotide polymorphisms (SNPs). By comparing the genetic structure and phenotypic variation among three genetic subpopulations, seven geographic distributions and four breeding periods, we found that geographic distribution and breeding period were not the determinants of genetic structure. In addition, no obvious phenotypic differentiations were found among the three subpopulations, even though they had different genetic backgrounds. A total of 324 SNPs and 160 candidate quantitative trait loci (QTL) regions were identified as significantly associated with the 16 agronomic traits. A network was established for multieffects in QTLs and interassociations among traits. Thirty‐eight associated regions had pleiotropic effects controlling more than one trait. One candidate gene, *Gh_D08G2376*, was speculated to control the lint percentage (LP). This GWAS is the first report using high‐resolution SNPs in upland cotton in China to comprehensively investigate agronomic traits, and it provides a fundamental resource for cotton genetic research and breeding.

## Introduction

Cotton (*Gossypium* spp.) is an economically important crop around the world, providing the most natural fibre for the manufacture of textiles. *Gossypium* contains more than 50 recognized species belonging to eight genome groups (Wendel and Grover, 2015). Only four species, *G. herbaceum* (A_1_), *G. arboreum* (A_2_), *G. hirsutum* (AD_1_) and *G. barbadense* (AD_2_), have been domesticated and cultivated. *G. hirsutum* L. (2*n* = 4*x* = 52, genome size: 2.5 Gb; Li *et al*., 2014a, 2015; Wendel and Grover, 2015), also called upland cotton, is cultivated worldwide and accounts for more than 95% of cotton production (Chen *et al*., 2007; Zhang *et al*., 2008). China is one of the largest cotton‐producing and cotton‐consuming countries in the world. The total production of cotton around the world has seen a decline in the past few years, but the consumption has increased annually (USDA, Cotton: World Markets and Trade, www.usda.gov). In addition, high‐end textile products using natural fibre have become increasingly popular. Therefore, it is important to improve the yield and fibre quality concurrently. In recent years, extensive studies have focused on forward and reverse genetics molecular biology methods to reveal genes for traits in upland cotton.

The vast majority of agronomic traits in cotton, as quantitative characteristics, are controlled by multigenes with small effects. Quantitative trait loci (QTL) mapping is an effective tool that has been widely used to dissect the genetic architecture of complex quantitative traits. It can also be effectively used in marker‐assisted selection (MAS) in cotton breeding. Traditional molecular markers, such as restriction fragment length polymorphism (RFLP), amplified fragment length polymorphism (AFLP) and simple sequence repeat (SSR), have played important roles in previous QTL mapping in cotton. Based on biparental recombinant populations, large numbers of intraspecific and interspecific genetic linkage maps have been developed and used in QTL mapping by linkage analysis in cotton (Fang *et al*., 2014; Jamshed *et al*., 2016; Wang *et al*., 2015a; Yu *et al*., 2014; Zhang *et al*., 2013). Limited by low map density and genetic diversity, linkage analysis can scan only a few QTLs and place the QTLs in a wide region (Cavanagh *et al*., 2008; Mackay and Powell, 2007). The use of high‐throughput technology, such as single‐nucleotide polymorphism (SNP) arrays and sequencing, can improve the resolution of genetic maps and the accuracy of QTL mapping. High‐density genetic maps (HDGMs) containing a large number of SNPs or insertion–deletion polymorphism (InDel) markers have been developed based on mixed‐type markers, SNP arrays or specific locus‐amplified fragment sequencing (SLAF‐seq) (Hulse‐Kemp *et al*., 2015; Wang *et al*., 2015b; Yu *et al*., 2012; Zhang *et al*., 2016). Combined with the published physical maps of *Gossypium* spp. (Li *et al*., 2014a; Li *et al*., 2015; Wang *et al*., 2012a; Zhang *et al*., 2015), these tools provide the capacity for fine mapping, candidate gene identification, gene functional exploration and MAS (Ma *et al*., 2016; Zhu *et al*., 2016).

Genome‐wide association study (GWAS) is an alternative tool for detecting QTLs and a promising genetic method for the dissection of complex traits in plants (Remington *et al*., 2001; Saidou *et al*., 2014). Compared to biparental linkage mapping, GWAS has the advantages of high resolution, cost‐efficiency and no necessity for creating a mapping population. GWAS has been widely used in studies of different plants, especially in crops such as maize, rice and oilseed rape. Based on a huge database of variant phenotype and high‐density genotypes covering the whole genome, GWAS is efficient and powerful for dissecting genetic variations and estimating their effects on phenotypes. Considering the interference of population attributes and environmental variation, computing models, such as general linear models (GLMs), mixed linear models (MLMs) and the Anderson–Darling test (AD test), have been developed using population structure, kinship or other factors as concomitant variants to reduce errors (Liu *et al*., 2016; Yang *et al*., 2014). In recent years, researchers have identified multiple marker loci associated with plant type, fibre quality, yield and resistance by association mapping using SSR markers (Abdurakhmonov *et al*., 2009; Liu *et al*., 2015a; Nie *et al*., 2016). However, the marker density and the population representation in these studies were limited.

The first 63K cotton array (CottonSNP63K Array) and a cotton HDGM based on this array were developed and published in 2015 (Hulse‐Kemp *et al*., 2015). In the present study, based on an extensive collection of 503 inbred *G. hirsutum* accessions, GWAS was performed using phenotypic data systematically investigated in a multiplot demonstration for 2 years and a genotypic data set of 11 975 SNPs from the HDGM of *Gossypium* spp. The aims of this study were to (i) clarify the genetic structure and linkage disequilibrium (LD) level of upland cotton in China, (ii) explore the genetic architecture of 16 agronomic traits and (iii) provide information about candidate QTL regions and genes that control the corresponding traits.

## Results

### Genotyping and genotypic statistical analysis

The 503 inbred upland cotton accessions were genotyped using the CottonSNP63K chip with a 63 058 SNP array. There were 61 928 (98.21%) SNPs reserved when the call rate was set at 90%. In addition, 31 069 (49.27%) polymorphic SNPs were screened out by the method of deleting the SNPs with minor allele frequencies (MAFs) <5%. After further exclusion, 11 975 SNPs showed high polymorphism and genotyping quality by match‐loading with the HDGM that contained 19 191 SNP markers. These SNP markers were used to assess population structure, relative kinship and GWAS analysis and covered 3854.3 cM of the entire cotton genome with an average density of one SNP per 0.32 cM, with the average distance ranging from 0.20 cM (Chr24) to 0.59 cM (Chr21) among different chromosomes. The average polymorphism information content (PIC) of all SNP markers was 0.332, varying from 0.279 (Chr08) to 0.369 (Chr01). In addition, the gene diversity varied from 0.32 (Chr08) to 0.44 (Chr01), with an average value of 0.39 for the entire genome (Table 1).

**Table 1:** Summary of SNPs, PIC, gene diversity and LD decay

#Chr	Chr length (cM)	#SNPs	SNP density (cM/SNP)	PIC	Gene diversity	LD decay (cM)	
						*r* ^2^ = 0.1	*r* ^2^ = 0.2
Chr01	144.31	471	0.31	0.369	0.44	5.0–5.5	4.5–5.0
Chr02	132.70	273	0.49	0.346	0.41	>10	>10
Chr03	138.72	388	0.36	0.339	0.40	3.5–4.0	0.5–1.0
Chr04	109.21	194	0.56	0.343	0.41	7–7.5	0.3–0.5
Chr05	210.41	677	0.31	0.348	0.41	>10	2.0–2.5
Chr06	136.60	345	0.40	0.309	0.37	7.0–7.5	5.5–6.0
Chr07	150.20	401	0.37	0.341	0.40	4.5–5.0	1.0–1.5
Chr08	171.00	852	0.20	0.279	0.32	5.0–5.5	1.0–1.5
Chr09	145.63	375	0.39	0.351	0.41	>10	0.5–1.0
Chr10	155.94	465	0.34	0.344	0.40	8.5–9.0	7.5–8.0
Chr11	191.73	483	0.40	0.297	0.35	5.0–5.5	1.5–2.0
Chr12	151.53	436	0.35	0.300	0.35	>10	0.5–1.0
Chr13	148.29	614	0.24	0.354	0.42	7.5–8.0	2.0–2.5
Chr14	160.44	567	0.28	0.359	0.43	5.0–5.5	1.0–1.5
Chr15	117.21	508	0.23	0.330	0.39	5.0–5.5	4.5–5.0
Chr16	133.55	509	0.26	0.354	0.42	6.5–7.0	2.5–3.0
Chr17	107.38	320	0.34	0.318	0.37	3.0–3.5	0.5–1.0
Chr18	125.72	394	0.32	0.310	0.36	5.5–6.0	0.5–1.0
Chr19	225.40	659	0.34	0.312	0.37	>10	0.5–1.0
Chr20	151.80	408	0.37	0.342	0.40	4.5–5.0	1.0–1.5
Chr21	184.43	310	0.59	0.316	0.37	7.0–7.5	2.0–2.5
Chr22	127.75	278	0.46	0.322	0.38	8.5–9.0	1.5–2.0
Chr23	127.11	395	0.32	0.356	0.42	4.5–5.0	0.5–1.0
Chr24	129.58	656	0.20	0.332	0.39	5.0–5.5	1.5–2.0
Chr25	131.29	556	0.24	0.347	0.40	>10	8.0–8.5
Chr26	147.42	441	0.33	0.335	0.39	2.5–3.0	1.0–1.5
Whole genome	3854.30	11 975	0.32	0.332	0.39	~6.1	~2.2

### Population LD decay, structure and kinship

**Figure 1:**
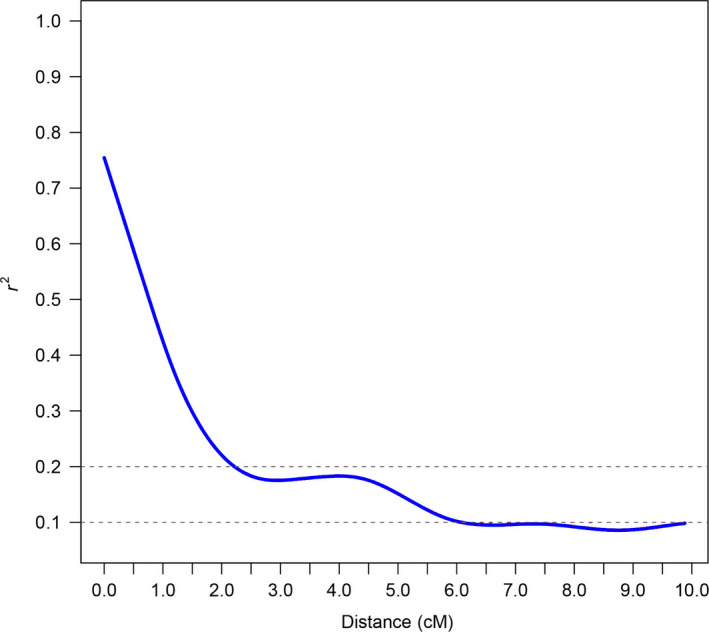
Linkage disequilibrium decay determined according to squared correlations of allele frequencies (*r* ^2^).

In this study, squared correlations of allele frequencies (*r* ^2^) were used to investigate the extent of LD calculated within a 0–10 cM window. The LD decay distance for the 503 accessions between all SNP markers was ~6.1 cM when the value of the cut‐off for *r* ^2^ was set at 0.1 and was ~2.2 cM when the value of the cut‐off for *r* ^2^ was set at 0.2 (Figure 1). In addition, the LD decays were not evenly distributed among chromosomes (Table 1; Figure S1), which ranged from 0.3–0.5 cM (Chr04) to 8.0–8.5 cM (Chr25) when *r* ^2^ = 0.2, or ranged from 2.5 to 3.0 cM (Chr26) to more than 10 cM (Chr02, Chr05, Chr09, Chr12, Chr19 and Chr25) when *r* ^2^ = 0.1. Four pairs of homologous chromosomes, Chr01‐Chr15 (5.0–5.5 cM), Chr03‐Chr17 (3.5‐4.0 cM), Chr05‐Chr19 (>10 cM) and Chr08‐Chr24 (5.0–5.5 cM) (*r* ^2^ = 0.1), showed similar LD decays. However, obvious LD differentiation, defined as a difference in the two LD decay distances >3 cM, was found among five pairs of homologous chromosomes: Chr02‐Chr14, Chr06‐Chr025, Chr09‐Chr23, Chr10‐Chr20 and Chr12‐Chr26.

**Figure 2:**
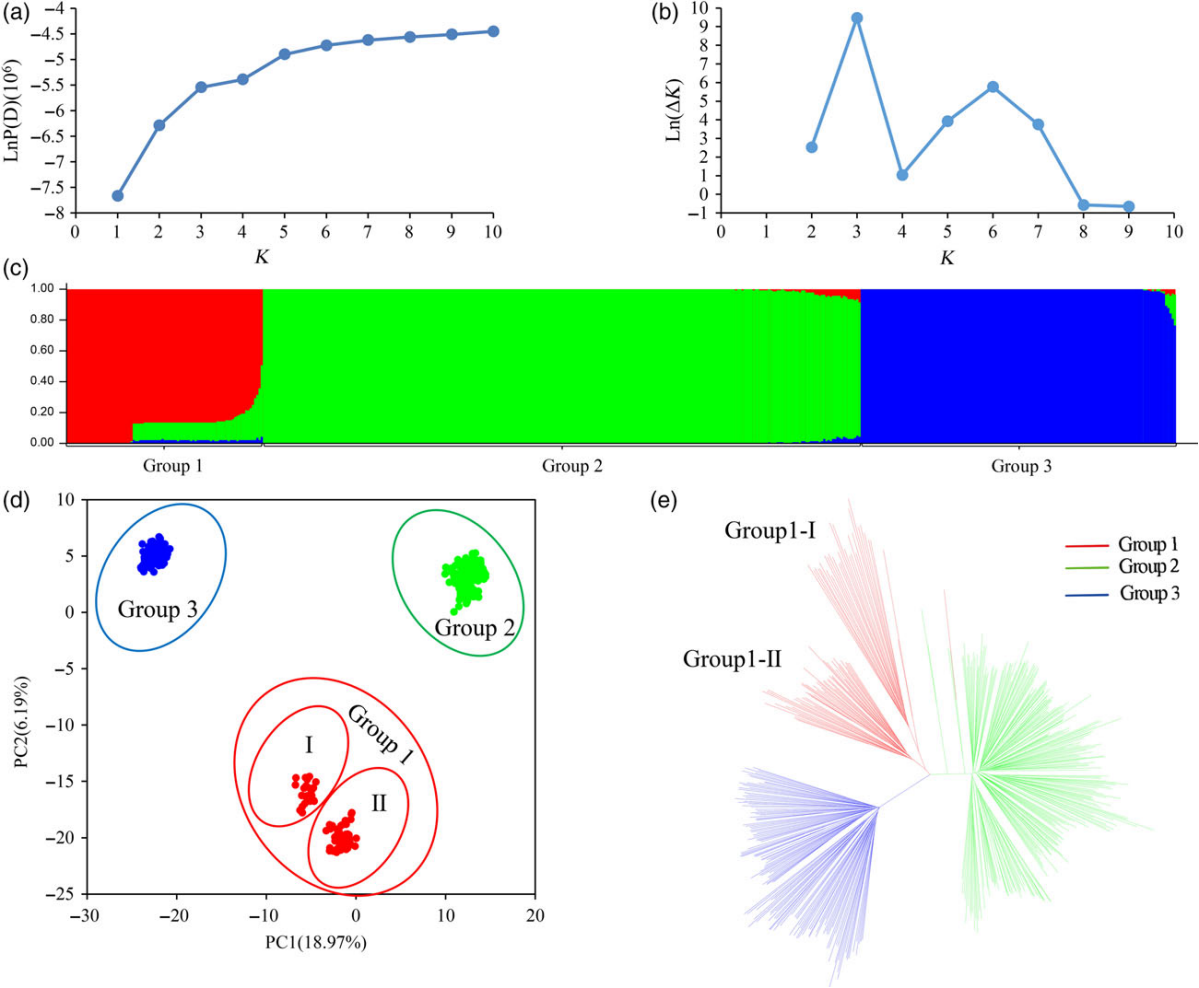
Population structure of the 503 accessions. (a) Mean LnP(D) values plotted from 1 to 10. (b) Ln(Δ*K*) values plotted from 1 to 10. (c) Population structure of the 503 accessions based on STRUCTURE when *K *= 3. (d) Principal component analysis of the 503 accessions based on genotype. (e) NJ tree based on Nei's genetic distances.

Because the population structure affects the authenticity of QTL mapping, understanding the structure matrix is of critical importance in GWAS populations. Three methods were used to estimate the number of subgroups in the 503 cotton accessions based on the genotypic database. First, STRUCTURE software was used to calculate the Bayesian clustering from *K *= 1 to 10 for five repetitions. The value of LnP(D) increased continuously from *K *= 1 to *K *= 10 without an obvious inflexion point (Figure 2a). However, the value of Evanno's Δ*K* showed an obvious spike at *K *= 3 (Figure 2b). It was suggested that the population could be divided into three subgroups (Figure 2c). A subset of 370 accessions (73.55%) showed pure components. In addition, 124 accessions (24.65%) consisted of <20% mixed subpopulations, and only nine accessions (1.78%) showed more than 20% admixture. Second, the genotypic principle component analysis (G‐PCA) revealed that the top three eigen‐vectors, PC1 (18.97%), PC2 (6.19%) and PC3 (5.44%), accounted for more than 30% of the genetic variation. The PC1–PC2 spatial distribution map showed that three subpopulations were clearly separated without overlapping regions (Figure 2d). Third, a neighbor‐joining (NJ) phylogeny based on Nei's genetic distances also showed that the 503 accessions were outlined with three main clusters (Figure 2e). Overall, the consistent results of STRUCTURE, PCA and the phylogeny tree confirmed that there were three subpopulations in the population panel.

Three subpopulations, defined as Group 1, Group 2 and Group 3, contained 89, 271 and 143 accessions, respectively (Tables S1‐S2). The genetic components of certain individuals in Group 1 were admixed from Group 2 (Figure 2c). This result suggested that there were two subgroups (Group 1‐I and Group 1‐II) in Group 1 (Figure 2c–e). Group 1‐II was generated through introgression from Group 2. The 503 accessions were also divided into seven groups by geographic origin or four groups by breeding stage (Table S1). Combined with the information from different clustering methods, it was found that three subpopulations of the 503 *G. hirsutum* accessions had no classified relationships with their geographic origin and breeding stage (Table S2; Figure S2). However, each of seven geographic origins and four breeding stages consisted of accessions from the three subpopulations. The accessions of seven geographic origins showed scattered mini‐gatherings on the round cluster tree (Figures S2, S3). Furthermore, the population genetic differentiation (*F*_ST_) of three subpopulations was greater than that of the clusters divided by breeding stage and geographic origin. The *F*_ST_ values ranged from 0.19 to 0.41 (three subpopulations), −0.09 to 0.02 (four breeding stages) and −0.04 to 0.09 (seven geographic origins; Table S3). The negative *F*_ST_ values represented that the average number of pairwise differences between two individuals from the intragroup was greater than from the intergroup.

**Figure 3:**
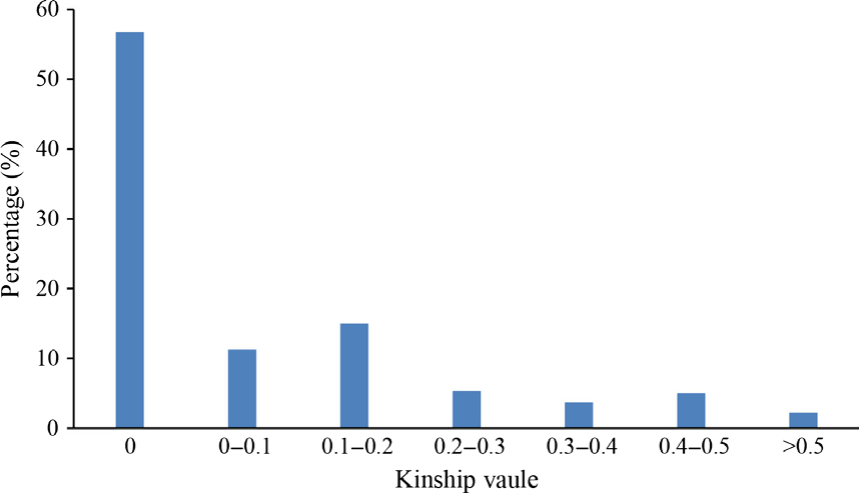
Histogram frequency distribution of pairwise relative kinship coefficients.

The kinship (K) matrix is another important factor for GWAS. The average pairwise relative kinship coefficient was 0.084. Pairwise relative kinship values of 0 accounted for 56.13% of all kinship coefficients. In addition, kinship values from 0 to 0.2 accounted for more than 80% of all pairwise kinship coefficients (Figure 3). Only 1.08% of the pairwise relative kinship coefficients were greater than 0.5. This result suggested that the 503 accessions were distantly related in this study.

### Phenotypic statistical analysis

The 16 traits were phenotyped and BLUPed with a multienvironment evaluation (Table S4). The *h* ^2^ ranged from 46.1% (fruit spur branch number, FSBN) to 91.8% (fibre strength, FS), with an average *h* ^2^ of 78.9%. The coefficient of variation (CV) ranged from 0.79% (fibre uniformity, FU) to 12.33% (first fruit spur height, FFSH) (Table S5). An analysis of variance (ANOVA) was used to reveal the effects of G (genotypes), E (environment) and G × E (interaction between G and E) for the 16 traits in multienvironments. Significant variations (*P *< 0.001) were observed in G and E. This result suggested that both the genotype and environment had important effects on these traits. In addition, the main effects were decided by the genotype. Furthermore, significant variations were observed in six traits in G × E by the *P* value <0.001 (WGP: whole growth period, FSBN, LP and MV: micronaire value) or *P* value <0.05 (FP: flowering period and EBN: effective boll number) (Table S6). The six traits were controlled by the genetic, environment effect and their interaction, and the former played the dominant role.

PCAs and statistical comparisons were used to explore the interassociations between phenotypes and clusters. The top two eigenvectors of phenotypic PCA (P‐PCA), PC1 and PC2, accounted for 74.9% of the phenotypic variation. The P‐PCA scatterplot illustrated that the spots for Group 1, Group 2 and Group 3 overlapped with each other. The four breeding stages and seven geographic origins had recognizable differential distributions among different groups (Figure S4a–c). In addition, the statistical analysis and LSD ANOVA showed that the phenotypes of the 16 traits were not significantly different among pairs of three groups (Table S7). However, the 16 traits, except seed‐cotton weight (SW) and lint weight (LW), showed significant discrepancies between each pair of the three major cotton belts in China (the Yangtze River Region (YtRR), the Yellow River Region (YRR) and the Northwestern Inland Region (NIR)) (*P *< 0.01 or 0.05). The 16 traits, except FSBN, SW and FE (fibre elongation), showed significant discrepancies between each pair of the four breeding stages (Table S7c). Moreover, the phenotypic values for FP, WGP, FFSBN (first fruit spur branch number), EBN, LW, LP, SF and FE distributed in YtRR, YRR and NIR gradually decreased from the south to the north of China, while FFSH, FSBN, FUHML (fibre upper half mean length), FS and FU distributed in an increasing tendency. Additionally, the statistics for FSBN, EBN, LW, LP, FUHML, FS and FU were increased from S1 to S4, while SF was decreased (Table S7b). This result revealed that the typical traits of yield and fibre quality have been effectively improved over the past years.

### Marker–trait GWAS

Considering the false positive in trait–marker associations, six common models for association analysis (the naive model (GLM), the Q model (GLM (Q)), the PCA model (GLM(PCA)), the K model (MLM(K)), the PCA + K model (MLM(PCA + K)) and the MLM model (Q + K)) were compared using a quantile–quantile (Q‐Q) plot (Figures S5b–S20b), and the best model was selected. For the 16 traits, the MLMs (K, PCA + K and Q + K models) were significantly better than the GLMs (naive, Q and PCA models). The Q–Q plot based on the naive GLM and Q models deviated from expectation, as shown in Figures S5b‐S20b. In the three MLMs, the Q + K and PCA + K models, controlling both population structure and relative kinship, were better than the K model, which only reduced errors in kinship. In this study, the Q + K model was chosen for the GWAS analysis.

The association analysis was based on BLUPed traits and 11 975 SNP markers. Significantly associated SNPs were detected for all the traits using different models at −lg(*p*) > 4.078 (Bonferroni correction *P *<* *1/11 975). A total of 324 significant SNPs were detected for all traits using the Q + K model (Tables 2, S8–S9). In addition, 290, 305, 244, 286 and 143 of the 324 SNPs were validated in the naive GLM, Q, PCA, K and PCA + K models, respectively, suggesting the reliability and repeatability of the Q + K model (Table S8). The significant SNPs identified for the 16 traits ranged from 1 (EBN) to 76 (FP), while the *R* ^2^, the phenotypic variation explained by SNPs, ranged from 3.17% to 9.04%, with an average of 4.48%. Given that the LD decay distance was set as a confidence interval for a QTL, a total of 160 QTLs were identified from the 324 SNPs (Table 2, Table S10). Moreover, 81 QTLs were also identified by the PCA + K model (Table S10). The candidate loci ranged from 1 (EBN) to 23 (FP) among the 16 traits, while it ranged from 2 (Chr02 and Chr03) to 13 (Chr20) among the 25 chromosomes (Table S11). Chr18 did not contain any QTLs. A total of 55 loci identified two or more significant SNPs, such as 30 SNPs located in *qGhFP‐c17‐1*. Except for EBN, all of the traits were scanned at multiple loci, and seven traits, FP, WGP, plant height (PH), LW, LP, FS and SF, were identified at more than 10 loci. According to the correspondence between the genetic and genome maps, 158 of 160 QTLs were assigned on physical regions with an average length of 6.36 Mb using BLAST, and a total of 29 390 genes were located in the riveted regions (Table S10). The genes were unevenly distributed in QTL physical regions ranging from 18 to 1038, with an average of 186 per loci. In addition, eight SSR markers identified with significant associations in the same population (Nie *et al*., 2016) were located in the related trait loci in this study (Table S12).

**Table 2:** Summary of significant SNPs by MLM (Q + K)

Trait	# SNP	# QTL	−lg(*p*) range	Average *R* ^2^ (%)	*R* ^2^ (%) range
FP	76	23	4.10–9.34	4.63	3.19–9.04
WGP	65	21	4.09–8.44	4.63	3.20–8.15
FFSH	15	5	4.12–6.26	4.54	3.23–6.23
FFSBN	13	9	4.10–5.98	3.98	3.18–5.68
PH	20	10	4.16–6.70	4.44	3.31–5.63
FSBN	3	3	4.15–5.02	3.66	3.23–4.01
EBN	1	1	4.56–4.56	3.59	3.59–3.59
SW	3	3	4.15–5.71	4.82	3.92–5.41
LW	28	22	4.10–6.17	4.28	3.23–5.89
LP	21	13	4.10–6.03	4.34	3.58–5.73
FUHML	11	7	4.32–5.79	4.36	3.44–4.88
FS	20	10	4.10–6.95	4.39	3.17–6.62
MV	4	3	4.12–4.85	3.65	3.41–3.94
FU	12	8	4.12–4.96	4.07	3.57–4.55
SF	26	19	4.09–7.71	4.55	3.35–7.37
FE	6	3	4.37–7.15	5.27	3.42–6.88
All traits	324	160	4.09–9.34	4.48	3.17–9.04

FP, flowering period; WPG, whole growth period; FFSH, first fruit spur height; FFSBN, first fruit spur branch number; FSBN, fruit spur branch number; PH, plant height; EBN, effective boll number; SW, seed‐cotton weight; LW, lint weight; LP, lint percentage; FUHML, fibre upper half mean length; FS, fibre strength; MV, micronaire value; FU, fibre uniformity; SF, short fibre, FE, fibre elongation.

For different traits, one QTL had a variable effect. For example, the allele ‘GG’ of i08818Gh (Chr05, 205.7 cM) had positive effects on FP (2.1%) and WGP (3.2%), but a negative effect on LP (−0.9%). The QTL effect for population phenotype was defined as the average of the cumulative allele effect. Interestingly, the QTL effects were discrepant among Group 1, Group 2 and Group 3. In contrast, there were minor relative differences in QTL effects among S1, S2, S3 and S4 and YtRR, YRR and NIR (Table S13; Figure S21).

### Pleiotropy and network of QTLs

**Figure 4:**
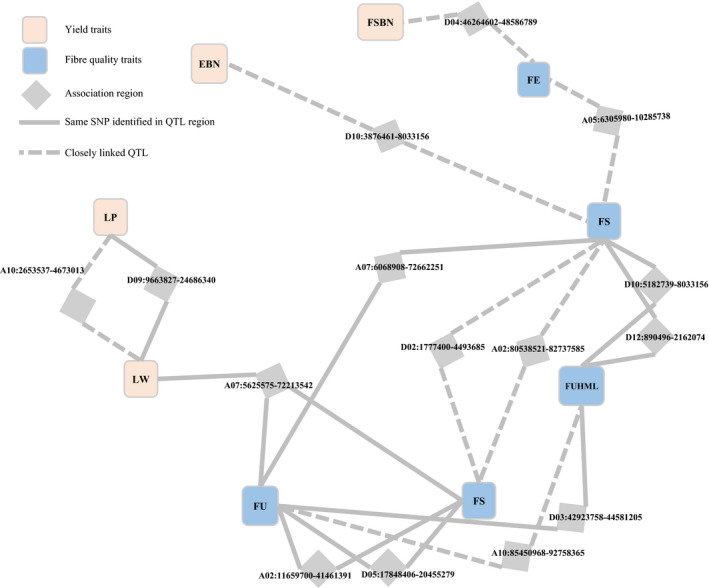
Network‐dissected QTL regions containing associations for yield and fibre quality traits. FSBN, fruit spur branch number; SW, seed‐cotton weight; LW, lint weight; LP, lint percentage; FUHML, fibre upper half mean length; FS, fibre strength; FU, fibre uniformity; SF, short fibre; FE, fibre elongation.

Significant correlations between traits were due to gene linkage or pleiotropy. Twenty‐eight QTL regions with significant SNPs were associated with more than one trait (Figures 4, S22–S23). Six of them, A02: 11 659 700–41 461 391; A05: 167 252–2 076 107; A07: 11 242 836–16 923 805; D03: 30 561 812–39 779 830; D10: 54 168 403–57 227 154; and D12: 41 604 652–44 320 641, were mapped to three traits (Figures 4, S23). In addition, 11 regions were closely linked (< 6.1 cM) with different loci (Figure S23). For example, *qGhPH‐c10* and *qGhLW‐c10‐1* were located at the close loci of ~76.16 and 76.55–76.6 cM, respectively. The 39 regions covered 84 QTLs and constructed association networks for different traits. In most cases, the regions linked to multiphenotypic traits were found among the same types of traits, that is growth period‐related (FP, WGP, FFSH and FFSBN), yield (FSBN, EBN, SW, LW and LP) or fibre quality traits (FUHML, FS, FU, FE, MV and SF). For example, there were nine QTL regions related to two growth period traits, FP and WGP. Twenty‐three regions were associated with the same type traits, and 12, 9 and 2 for growth period‐related, fibre quality and yield traits, respectively. However, several QTL regions were detected that contained significant associations for different types of traits. Four regions were identified with associations for multiple traits between fibre quality and yield traits (Figure 4). However, three and seven regions were observed with intertype pleiotropy, respectively, linked to growth period–yield and growth period–fibre quality traits (Figure S23). This finding suggested that QTL networks govern agronomic traits with multiple effects and span space‐time as a whole.

### Candidate gene finding

**Figure 5:**
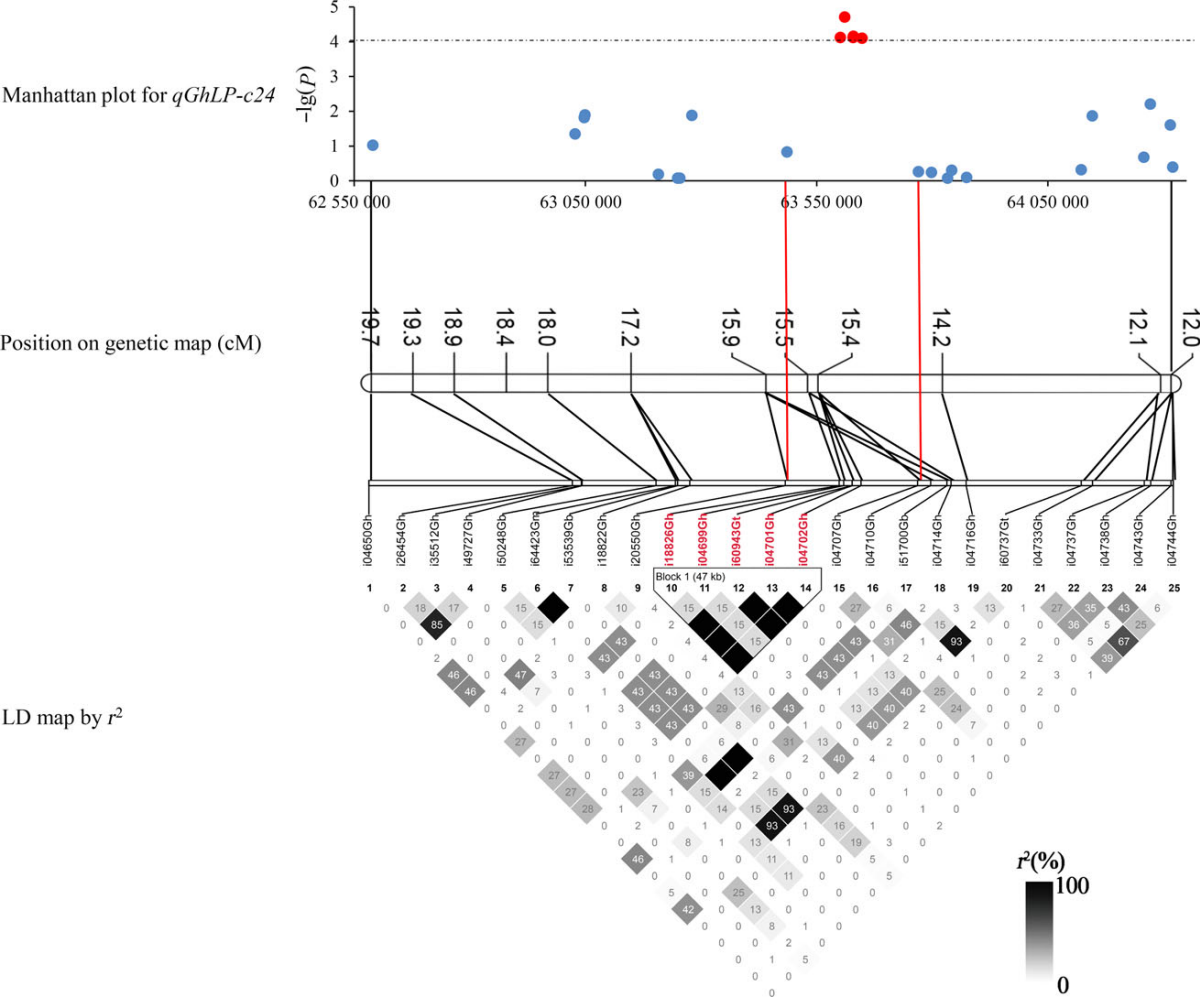
*qGhLP‐c24* on Chr24 (D08: 62 589 986–64 319 659) was associated with lint percentage. Manhattan plot shown for the D08: 62 589 986–64 319 659 region. The red plot and the SNPs were significantly associated with LP.

The LD decay set as a confidence interval was used to identify candidate genes. In general, in the QTLs that covered candidate genomic regions with narrow peaks, it was easy to find candidate genes. However, most loci covered regions containing dozens of genes or a few significant SNPs. There were only eight association regions including 12 QTLs with each one covering more than five significant SNPs. Only two of the eight regions, *qGhLP‐c24* and *qGhSF‐c20*, had narrow LD decay distances of less than 1 Mb at *r* ^2^ < 0.1; the others had LD decay distances of more than 5 Mb. The LD decays around *qGhLP‐c24*, assigned on D08 (62 589 986–64 319 659) using BLAST, were 450 kb and 150 kb when *r* ^2^ = 0.1 and 0.2, respectively. The *qGhLP‐c24* accounted for 3.99% of the variation in LP, and the minimum *P*‐value was 0.000076. Combining the LD heatmap, the candidate region was finally focused between two SNPs, i20550Gh (63 485 399) and i04707Gh (63 648 326) (*r* ^2^ = 0.2; Figure 5), and 17 genes were identified in this region (Table S14). Twelve genes were annotated in *Arabidopsis thaliana*, two of which, *Gh_D08G2369* and *Gh_D08G2376*, were preferentially expressed in fibre.

From the TM‐1 gene expression database, a number of genes had specific expression patterns in the tissues, with typical spatial organ or development stages in cotton, such as root, stem, leaf, anther, stigma, ovule, fibre [10 and 20 days postanthesis (DPA)] and seed (10 DPA and 20 DPA). These genes were supposed to affect related traits; for example, the genes preferentially expressed in fibre may be involved in fibre yield and quality. After screening, 336 genes were obtained and were hypothesized to be the most likely candidate genes for 86 QTLs (Tables S15–S16). There were 114 genes preferentially expressed in root, stem and leaf located in the QTL region associated with the growth period traits PH and FSBN, which are mostly shaped by the vegetative growth. The gene expression profiles of the boll yield traits BW, LW and LP were focused in the ovule, fibre and seed, and 169 genes were selected out. In addition, 53 genes preferentially expressed in 10 DPA and 20 DPA fibre were noted as candidates for fibre quality traits.

The genes with known functions could also afford evidence for candidate gene identification. Nineteen known genes that had been reported to function in cotton fibre were located in 25 identified QTL regions using BLAST (Table S17). In detail, 11 and nine genes were identified in the loci for boll yield‐related (SW, LW and LP) and fibre quality traits, respectively. In addition, two GAST (GA‐stimulated transcript)‐like genes, *GASL3* and *GhGASL5*, were riveted on D04 loci associated with FSBN and FP. Several genes were on more than one site and located in the QTL that controlled different traits. For example, *GhACT1* has three loci that control SF or LW. The functions of 19 genes could explain the corresponding traits directly or indirectly. For example, four *GhGASLs* located in five QTL regions that controlled FSBN, FP, SF and LW were reported as targets of GA regulation that could control seed germination and storage mobilization, stem elongation, flower initiation, pollen and fruit growth and root development.

## Discussion

### Population and SNP markers

GWAS has been used to map complex quantitative traits in plants (Atwell *et al*., 2010; Crowell *et al*., 2016; Ingvarsson and Street, 2011; Lu *et al*., 2015; Zhao *et al*., 2011). The power of GWAS mainly depends on four factors: the richness of genetic diversity, the veracity of trait acquisition, the marker density and the statistical methods. Our *G. hirsutum* cultivar collection from China has high levels of genotypic and phenotypic diversity. The 503 accessions cover the five cotton‐growing regions in China. A few accessions were introduced from the Soviet Union (SU) and the United States (USA), such as Deltapine, Coker and Stoneville, which are the founder accessions for cotton breeding in China and have made significant contributions to Chinese cotton production. The relatively larger sample size ensured sufficient genetic variation, and the sample size was similar to the GWAS population sizes used for *Arabidopsis thaliana* (Zhao *et al*., 2005), *Oryza sativa* (Crowell *et al*., 2016; Famoso *et al*., 2011), *Zea mays* (Li *et al*., 2013a; Wen *et al*., 2014) and *Brassica napus* (Xu *et al*., 2016).

The phenotypic variance associated with the environment disturbs the reliability of QTL mapping. Multienvironment programmes and unbiased predictions are practical ways to correct for this error. The four sites for the trait experiments—Huanggang, Hubei Province (HG, E114.87°, N30.44°); Yuanyang, Henan Province (YY, E113.97°, N35.05°); Shihezi, Xinjiang (SHZ, E85.94°, N44.27°); and Korla, Xinjiang (KRL, E86.06°, N41.68°)—are located in the three main cotton belts in China. In addition, the four sites belong to four climate classifications, Cfa (warm temperate and fully humid), Cwa (warm temperate and desert), BSk (cold and arid steppe) and BWk (cold and arid desert), respectively (Chen and Du, 2006). There are large differences in geographic position and climate among these sites. The phenotyping from multiple plots and years effectively eliminated the influence of the environment. The 16 agronomic traits possessing abundant phenotypic variation and stable heritability (Tables S5–S6) are suitable to reveal their genetic basis.

The SNP array approach is reliable, efficient and high throughput for genotyping. The CottonSNP63K array, the first SNP chip for cotton, was developed from discovery sets that represent a diverse range of *G. hirsutum* germplasm, including *G. hirsutum*,* G. barbadense*,* G. tomentosum*,* G. mustelinum*,* G. armourianum* and *G. longicalyx* (Hulse‐Kemp *et al*., 2015). This array showed high polymorphism (49.27%) in an upland cotton panel compared with biparental recombinant populations or SSR markers (Li *et al*., 2016; Wang *et al*., 2015b). The average density of polymorphic SNPs was 1SNP/0.32 cM, equal to ~200 kb in the physical map, which achieved the requirement for GWAS mapping. In previous studies in other plants, it has been the preponderant trend to use SNP arrays and high‐density mapping with more than 10 000 SNPs for GWAS in cotton.

### Population structure of upland cotton in China

The population structure is important for explaining the heterogeneity of genetic architecture and is mostly affected by geographic isolation and gene exchange isolation. The panel of 503 accessions covered seven regions [YtRR, YRR, NIR, the Northern Specific Early Maturation Region (NSEMR), the Southern China Region (SCR), USA and SU) and four breeding stages (S1, S2, S3 and S4]. The accessions were classified into three subpopulations (Group 1, Group 2 and Group 3) but were not completely separated according to geographic origin and breeding stage. Three major cotton belts, YtRR, YRR and NIR, composed 88.47% of the whole panel. Three similar genetic backgrounds were present among the groups of YtRR, YRR and NIR, along with S1, S2, S3 and S4 (Table S3; Figure S2). We demonstrate that interspersed introduction or crossbreeding of upland cotton germplasm led to gene exchange among different regions in China. The results also indicated a relatively open system for gene exchange liberated from geographic restrictions (Nie *et al*., 2016; Zhao *et al*., 2014). Chinese upland cotton breeding and production were based on the introduced germplasms and improved or cultivated species in recent decades (Chen and Du, 2006) based on founder germplasms with heritable population structures. We take the view that there are three subpopulations that are not affected by geographic factors and that are formed at the early breeding stage.

Several traits have been developed by environment adaptation or production requirements. Significant phenotypic differences were revealed in different regions and breeding stages. The high‐latitude cotton belts of China with shorter suitable seasons for growing set the upland cotton breeding objective to early maturity. The FP and WGP for the accessions from NIR are significantly earlier than those from YRR and YtRR. It was also found that fibre quality was better in the NIR (Table S7). In the four breeding stages, genetic diversity slightly decreased, and the advantageous economic characteristics significantly improved over time. Due to founder effects, the genetic resources are mainly inherited from cultivars introduced early, mainly in S1 and S2. Both the positive and the negative effect alleles were preserved during the process of breeding selection (Figure S21). However, three subpopulations (G1, G2 and G3) were identified based on genotypic data, which was evidence that there were different alleles and genetic effects among them (Table S13). However, the subpopulations with different genetic backgrounds did not result in significant differences in phenotypes (Table S7a). In the past breeding process, the same target, especially for high yield, was achieved through different combinations of genetic sources in each system. The more advantageous allelic genes gathered could yield better improvements in quantitative traits that were affected by the additive effects (Li *et al*., 2013a; Wang *et al*., 2006).

### LD in upland cotton of China

The speed of LD decay determines the resolution and capacity of marker–trait association mapping. According to previous studies, LD decay differs among species and populations. The attenuation distances in *Zea mays*,* Oryza sativa* and *Brassica napus* were <100 kb, <1 and <6 Mb, respectively (Crowell *et al*., 2016; Flint‐Garcia *et al*., 2003; Jamshed *et al*., 2016; Xu *et al*., 2016). In this study, upland cotton in China had a slower LD decay of 6.1 cM (~4 Mb) when *r* ^2^ < 0.1 in the whole genome. However, previous studies have found that the LD decay distance of upland cotton ranged from 3.4 to 25 cM, as estimated using SSR markers (Abdurakhmonov *et al*., 2008; Fang *et al*., 2013). It is unfavourable to find genes of traits at such LD levels in other species. Recombination, mutation, population bottlenecks, founder effects, drift, selection migration and population admixture are the main causes of LD (Mackay and Powell, 2007; Morrell *et al*., 2005). We speculated that the relatively low rate of outcrossing and the short breeding history of upland cotton in China may be the main reasons for the slower LD decay. It is also possible that genetic variation is lost due to inbreeding and the founder effect. In addition, we found different LD decays among different chromosomes (Table 1; Figure S1). The chromosomes with lower LD are hypothesized to have been involved in the domestication process (Li *et al*., 2013b). Thus, the higher LD chromosomes, such as Chr05, Chr09, Chr12, Chr19 and Chr25, should have experienced high‐frequency selection in breeding. Several pairs of homologous chromosomes had similar levels of LD, such as Chr01 (A01)‐Chr15 (D01), Chr03 (A03)‐Chr17 (D03) and Chr08 (A08)‐Chr24 (D08). In addition, the LD decay in the homologous chromosomes Chr05 (A05) and Chr19 (D05) was more than 10 cM in both cases. It is supposed that conserved or stabilized genetic recombination occurred during the breeding process on these homologous chromosomes.

### Efficiency of GWAS for complex agronomic traits

GWAS is an efficient method for QTL mapping agronomic traits in upland cotton. In previous studies, a number of loci were identified as associated with yield components and fibre quality using SSRs under the standard threshold at *P *< 0.05 or 0.01 (Abdurakhmonov *et al*., 2009; Nie *et al*., 2016; Qin *et al*., 2015). GLM and MLM are the two prevalent statistical models in association mapping. However, the MLM simultaneously estimates the population structure and kinship among individuals, allowing a large reduction in false associations (Wang *et al*., 2012b). In the current study, the MLM (Q + K) model was chosen after comparing six models using phenotype–genotype association analysis. Detected by the MLM (Q + K), a total of 160 QTLs were found that distributed over 26 chromosomes for 16 traits. In addition, MLM (Q + K) was a credible model for association studies; 94% and 87% of loci were shared with GLM (Q) and MLM (K), respectively. In addition, several SSR loci significantly associated with traits analysed in the same population (Table S12; Nie *et al*., 2016) were located in the QTLs controlling the same or related traits in this study. For example, MON‐CGR5867 and HAU1434, associated with FP, were located in *qGhFP‐c9‐2* and *qGhFP‐c12‐1*, respectively. Some reported genes with known functions were also detected in this study (Table S14). In our results, only three yield traits, FSBN, EBN and SW, mapped fewer QTLs than other traits. The lower heritability of these traits indicated that they were sensitive to the environment. In addition, the strong effect of the environment will reduce the efficiency of QTL mapping. Furthermore, the associated QTLs of all traits, detected with low explained phenotypic variation (*R* ^2^) (Table 2), revealed the general quantitative trait characteristics of minor and additive effects. GWAS is a powerful tool for mining the gene loci of the agronomic traits in upland cotton.

High‐resolution GWAS can quickly yield gene mapping and identification in cotton. Generally, candidate genes are screened in the confidence interval determined by LD decay. Unfortunately, the relatively high LD level in upland cotton of China creates some challenges to identify the exact candidate genes. To provide the reference gene, a list of *a priori* candidate genes involved in a preferential expression pattern or that have been clearly studied by cloning and characterizing were assembled. In this study, a candidate region containing 17 genes for LP was found, and the candidate gene *Gh_D08G2376* was suggested by in silico expression analysis (Table S14). Although there is no function information for *Gh_D08G2376* in cotton, its homologous genes *AT3G07020* and *GhSGT1* have clear function information. *AT3G07020* (*UGT80A2*) mutant plants have lower steryl glycoside and acyl steryl glycoside levels and reduced seed size in *Arabidopsis* (DeBolt *et al*., 2009; Stucky *et al*., 2015). The gene *GhSGT1* has high glucosylation activities for sitosterol‐glucoside products in cotton fibres (Li *et al*., 2014b). *Gh_D08G2376* may regulate seed size and fibre development and then affect the LP. These candidate genes can be studied in future verification studies. More experiments can be designed to quickly confirm the candidate genes, such as developing customized recombination by crossing to fine mapping and detecting the expression of genes in target regions, to find the connections between gene expression and phenotypic variation.

### Multicorrelation and QTL pleiotropism among traits

The phenotypes are affected by multigenes; in addition, one gene can decide multiphenotypes (Crowell *et al*., 2016), defined as the gene additive effect and pleiotropy, respectively. Given the relationship between traits and genes, 38 regions with significant QTLs for more than one trait were observed; six regions had associations for three traits. An example was the QTL in region D10: 54 168 403–57 227 154 controlled three agronomic traits, FP, WGP and FUHML, which are different in developmental space‐time (Figure 4). In general, the same types of traits had overlapping QTL regions in the genome, such as 12 regions associated with four growth period traits (FP, WGP, FFSH and FFSBN), nine regions associated with four fibre quality traits (FUHML, FS, FU and SF) and two regions associated with three yield traits (SW, LW and LP; Figures 4 and S23). Additionally, 18 overlapped or linkage regions were associated with different type traits; for example, four QTL regions linked fibre quality and yield traits (Figure 4). Organisms are tightly controlled systems that link with source–sink translocation and morphological traits. The mutual coordination of the system must include actual production. The QTL and traits network provides valuable information to explore inherent biological mechanisms among genes and traits in upland cotton.

## Conclusions

The 503 upland cotton accessions in China were genotyped using a 63K SNP array and clustered into three subpopulations via 11 975 polymorphic SNPs screened from the cotton HDGM. Three subpopulations, regarded as three independent subpopulations in the breeding programmes, were not correlated with geographies and breeding periods. The whole‐genome LD decay distance was 6.1 cM for this population. Based on MLM (Q + K), 324 significant SNPs and 160 QTLs were identified and associated with 16 agronomic traits. Pleiotropy and close connections were identified and established by a traits network. A number of genes are prospective candidate genes, and further work will be conducted to investigate them. Our study can be applied in genetic research and breeding in cotton.

## Experimental procedures

### Plant materials

In this study, a diverse collection of 503 *G. hirsutum* inbred accessions was assembled for genotyping and phenotyping. The collections were derived from five main China cotton belts: the YtRR (141), the YRR (225), the NIR (79), the NSEMR (28) and the SCR (4), and historically introduced varieties and germplasm resources lines from abroad: the SU (6) and the USA (20) (Nie *et al*., 2016). The 503 accessions were introduced or bred from 1901 to 2011, divided into four breeding stages: 1920s–1950s (S1, 21), 1950s–1980s (S2, 135), 1980s–2000s (S3, 184) and 2001s–2010s (S4, 163) (Table S1). All the accessions were authorized for scientific research purposes only.

### Phenotypic design and statistical analysis

A multiple‐environment experiment was designed for phenotyping to eliminate the environmental effect (Table S4). A total of 16 agronomic traits were investigated, including growth period, plant type, yield and fibre quality traits (Nie *et al*., 2016). ANOVA was performed to evaluate the effects of genotype (G), environment (E) and the interactions between genotype and environment (G × E). Best linear unbiased predictions (BLUPs) were used to estimate phenotypic traits across multiple environments based on a linear model. The statistical analyses of mean, standard error (SE) and LSD Duncan were calculated using IBM SPSS Statistics 21 (http://www.ibm.com/analytics/us/en/technology/spss/spss.html).

### SNP genotyping

The 503 accessions were grown in glasshouses for DNA sampling. Genomic DNA was extracted from the young leaf tissue. DNA was standardized at 50 ng/mL for each accession and was processed according to Illumina protocols. The DNA was then hybridized to the CottonSNP63K array, which contains 45 104 putative intraspecific SNP markers from the cultivated cotton species *G. hirsutum* and 17 954 putative interspecific SNP markers from other cotton species (Hulse‐Kemp *et al*., 2015). Using the CottonSNP63K array, Hulse‐Kemp *et al*. (2015) developed a high‐density mapping of intraspecific and interspecific populations of *Gossypium* spp. The genetic map contained 19 191 SNP markers mapped to 26 linkage groups that collectively encompass 3854.3 cM. The polymorphism markers were screened by the threshold that called rates >90% and minor allele frequency (MAF) >5%.

### Population structure, kinship and LD analysis

The population structure of the 503 accessions was analysed using a Bayesian Markov chain Monte Carlo (MCMC) model with STRUCTURE 2.3.4 software (Evanno *et al*., 2005). The number of simulation subgroups (*K* value) was set from 1 to 10. The natural logarithms of probability data (LnP(*K*)) and the *ad hoc* statistic Δ*K* were calculated using STRUCTURE HARVESTER: a website and program for visualizing STRUCTURE output and implementing the Evanno method (Earl and vonHoldt, 2011) (http://taylor0.biology.ucla.edu/structureHarvester/). The Δ*K* was set as the determinant factor for evaluating the optimal value of *K* (Mezmouk *et al*., 2011). The Q‐matrix was obtained after integrating the five replicate runs using CLUMPP software (Jakobsson and Rosenberg, 2007).

PCA performed using TASSEL version 5.0 (Bradbury *et al*., 2007) was also used to stratify the population structure. The PIC of the SNP markers, gene diversity and genetic distances among accessions were calculated using the software package PowerMarker version 3.25 (Liu and Muse, 2005). The NJ phylogenetic tree was plotted using Nei's genetic distances method (Nei 1972). The kinship (K) matrix was estimated using SPAGeDi version 1.4b (Hardy and Vekemans, 2002).

*F*_ST_ is a measure of population differentiation due to genetic structure and was calculated with PowerMarker version 3.25 using the genetic polymorphism data among different groups, clustered by genotypes, breeding stages and planting regions.

The parameter *r* ^2^, the correlation in frequency among pairs of alleles across a pair of SNP loci, was calculated with the software package TASSEL version 5.0.

### Genome‐wide association study

Phenotype–genotype association analysis and the allele effect calculations were performed using TASSEL version 5.0. Six models, a naive general linear model (GLM), the general linear model adjusted using the Q‐matrix (GLM (Q)) and PCA‐matrix (the top five principal components, GLM (PCA)), the mixed linear model adjusted using the K‐matrix (MLM (K)), the mixed linear model correcting for both Q‐matrix and K‐matrix (MLM (Q + K)) and the mixed linear model correcting for both PCA‐matrix and K‐matrix (MLM (PCA + K)), were employed to reduce errors from population structure and relative kinship. Bonferroni‐corrected thresholds at α  =  1 were set as the significance of associations between SNP markers and the traits (Liu *et al*., 2015b; Wang *et al*., 2012b). The threshold for the *P*‐value was set at −lg(1/*n*) when the number of SNP markers was *n*.

The interval of SNPs significantly associated with one trait less than the LD decay was labelled as one QTL region. The SNPs in QTL regions were selected out, and a BLAST (Altschul *et al*., 1994) search with *E* ≤ 1e−10 against the *G. hirsutum* genome (TM‐1, NAU‐NBI v1.1 assembly) was performed. Haploview 4.2 was used to generate an LD map based on physical location (Calati *et al*., 2011). Putative candidate genes were proposed for each locus using the gene annotation database at NCBI (http://www.ncbi.nlm.nih.gov/). More, the *G. hirsutum* gene expression database (Zhang *et al*., 2015) was used to identify the particular expressed genes in associated tissues of candidate regions.

## Conflict of interest

The authors declare no conflicts of interest.

## Supporting information


**Figure S1** Curve chart of linkage disequilibrium (LD) decay for each of the 26 chromosomes. (a) LD decay for 13 At‐genome chromosomes: Chr01‐Chr13. (b) LD decay for 13 Dt‐genome chromosomes: Chr14‐Chr26.
**Figure S2** Distribution of the three subpopulations in 7 region‐clusters and 4 breeding period‐clusters. (a) Percentages stacked column chart for 7 regions: YRR, YtRR, NIR, NSEMR, SCR, USA and SU. (b) Percentages stacked column chart for four breeding periods: S1, S2, S3 and S4.
**Figure S3** Round tree for the 503 accessions. The outer ring represents three subpopulations; the inner ring represents 7 regions.
**Figure S4** Principal component analysis of 503 accessions based on phenotype. (a) Scatter plot for three subpopulations: Group 1, Group 2 and Group 3. (b) Scatter plot for 7 regions: YRR, YtRR, NIR, NSEMR, SCR, USA and SU. (c) Scatter plot for four breeding periods: S1, S2, S3 and S4.
**Figure S5** Summary of GWAS results for flowering period (FP). (a) Phenotype histogram for FP. (b) Q‐Q plots for FP using GLM, GLM (Q), GLM (PCA), MLM (K), MLM (PCA+K), and MLM (Q+K). (c) Manhattan plot for FP GWAS results. The threshold value was set at *p *<* *10^−4.078^.
**Figure S6** Summary of GWAS results for the entire growth period (WGP). (a) Phenotype histogram for WGP. (b) Q‐Q plots for WGP using GLM, GLM (Q), GLM (PCA), MLM (K), MLM (PCA+K), and MLM (Q+K). (c) Manhattan plot for WGP GWAS results. The threshold value was set at *p *<* *10^−4.078^.
**Figure S7** Summary of GWAS results for first fruit spur height (FFSH). (a) Phenotype histogram for FFSH. (b) Q‐Q plots for FFSH using GLM, GLM (Q), GLM (PCA), MLM (K), MLM (PCA+K), and MLM (Q+K). (c) Manhattan plot for FFSH GWAS results. The threshold value was set at *p *<* *10^−4.078^.
**Figure S8** Summary of GWAS results for fruit spur branch number (FSBN). (a) Phenotype histogram for FFBN. (b) Q‐Q plots for FSBN using GLM, GLM (Q), GLM (PCA), MLM (K), MLM (PCA+K), and MLM (Q+K). (c) Manhattan plot for FSBN GWAS results. The threshold value was set at *p *<* *10^−4.078^.
**Figure S9** Summary of GWAS results for first fruit spur branch number (FFSBN). (a) Phenotype histogram for FFSBN. (b) Q‐Q plots for FFSBN using GLM, GLM (Q), GLM (PCA), MLM (K), MLM (PCA+K), and MLM (Q+K). (c) Manhattan plot for FFSBN GWAS results. The threshold value was set at *p *<* *10^−4.078^.
**Figure S10** Summary of GWAS results for plant height (PH). (a) Phenotype histogram for PH. (b) Q‐Q plots for PH using GLM, GLM (Q), GLM (PCA), MLM (K), MLM (PCA+K), and MLM (Q+K). (c) Manhattan plot for PH GWAS results. The threshold value was set at *p *<* *10^−4.078^.
**Figure S11** Summary of GWAS results for effective boll number (EBN). (a) Phenotype histogram for EBN. (b) Q‐Q plots for EBN using GLM, GLM (Q), GLM (PCA), MLM (K), MLM (PCA+K), and MLM (Q+K). (c) Manhattan plot for EBN GWAS results. The threshold value was set at *p *<* *10^−4.078^.
**Figure S12** Summary of GWAS results for seed weight (SW). (a) Phenotype histogram for SW. (b) Q‐Q plots for SW using GLM, GLM (Q), GLM (PCA), MLM (K), MLM (PCA+K), and MLM (Q+K). (c) Manhattan plot for SW GWAS results. The threshold value was set at *p *<* *10^−4.078^.
**Figure S13** Summary of GWAS results for lint weight (LW). (a) Phenotype histogram for LW. (b) Q‐Q plots for LW using GLM, GLM (Q), GLM (PCA), MLM (K), MLM (PCA+K), and MLM (Q+K). (c) Manhattan plot for LW GWAS results. The threshold value was set at *p *<* *10^−4.078^.
**Figure S14** Summary of GWAS results for lint percentage (LP). (a) Phenotype histogram for LP. (b) Q‐Q plots for LP using GLM, GLM (Q), GLM (PCA), MLM (K), MLM (PCA+K), and MLM (Q+K). (c) Manhattan plot for LP GWAS results. The threshold value was set at *p *<* *10^−4.078^.
**Figure S15** Summary of GWAS results for fibre upper half mean length (FUHML). (a) Phenotype histogram for FUHML. (b) Q‐Q plots for FUHML using GLM, GLM (Q), GLM (PCA), MLM (K), MLM (PCA+K), and MLM (Q+K). (c) Manhattan plot for FUHML GWAS results. The threshold value was set at *p *<* *10^−4.078^.
**Figure S16** Summary of GWAS results for fibre strength (FS). (a) Phenotype histogram for FS. (b) Q‐Q plots for FS using GLM, GLM (Q), GLM (PCA), MLM (K), MLM (PCA+K), and MLM (Q+K). (c) Manhattan plot for FS GWAS results. The threshold value was set at *p *<* *10^−4.078^.
**Figure S17** Summary of GWAS results for micronaire value (MV). (a) Phenotype histogram for MV. (b) Q‐Q plots for MV using GLM, GLM (Q), GLM (PCA), MLM (K), MLM (PCA+K), and MLM (Q+K). (c) Manhattan plot for MV GWAS results. The threshold value was set at *p *<* *10^−4.078^.
**Figure S18** Summary of GWAS results for fibre uniformity (FU). (a) Phenotype histogram for FU. (b) Q‐Q plots for FU using GLM, GLM (Q), GLM (PCA), MLM (K), MLM (PCA+K), and MLM (Q+K). (c) Manhattan plot for FU GWAS results. The threshold value was set at *p *<* *10^−4.078^.
**Figure S19** Summary of GWAS results for short fibre percentage (SF). (a) Phenotype histogram for SF. (b) Q‐Q plots for SF using GLM, GLM (Q), GLM (PCA), MLM (K), MLM (PCA+K), and MLM (Q+K). (c) Manhattan plot for SF GWAS results. The threshold value was set at *p *<* *10^−4.078^.
**Figure S20** Summary of GWAS results for fibre elongation (FE). (a) Phenotype histogram for FE. (b) Q‐Q plots for FE using GLM, GLM (Q), GLM (PCA), MLM (K), MLM (PCA+K), and MLM (Q+K). (c) Manhattan plot for FE GWAS results. The threshold value was set at *p *<* *10 ^−4.078^.
**Figure S21** Association loci effects in different clusters.
**Figure S22** Pleiotropic effects of GWAS QTLs. Orange represents overlapped QTLs for different traits, and blue represents the closely linked QTLs or different traits.
**Figure S23** Network‐dissected QTL regions containing associations for agronomic traits. (a) Growth period traits, plant height (PH) and yield traits. (b) Growth period traits, PH and fibre quality traits. FP: flowering period; WGP: whole growth period; FFSH: first fruit spur height; FFSBN: first fruit spur branch number; PH: plant height; LW: lint weight; LP: lint percentage; FUHML: fibre upper half mean length; FS: fibre strength; FU: fibre uniformity; SF: short fibre, FE: fibre elongation.

**Table S1** Information for 503 upland cotton accessions.
**Table S2** Number of accessions and gene diversity levels in different clusters.
**Table S3** Population genetic differentiation statistics (*F*_*ST*_) between pairwise clusters.
**Table S4** Data acquisition for sixteen traits from four places in 2012 and 2013.
**Table S5** Descriptive statistics for phenotypic variations and broad‐sense heritability for 16 traits.
**Table S6** ANOVA for 16 traits from multi‐environments in 2012 and 2013.
**Table S7a** Phenotypic comparisons among Group 1, Group 2 and Group 3 by ANOVA and LSD.
**Table S7b** Phenotypic comparisons among S1, S2, S3 and S4 by ANOVA and LSD.
**Table S7c** Phenotypic comparisons among YtRR, YRR and NIR by ANOVA and LSD.
**Table S8** Shared numbers of significant SNPs associated with 16 traits in six models.
**Table S9** The 324 significant SNPs associated with 16 traits.
**Table S10** Information for 160 QTLs and the number of genes in the QTL regions.
**Table S11** Number of significant SNPs and QTLs for traits and chromosomes.
**Table S12** The SSR markers identified in Nie's report located in our QTL regions.
**Table S13** Association loci effects in different clusters.
**Table S14** The 17 genes located in the candidate region of *qGhLP‐c24* and its annotation in *Arabidopsis thaliana*.
**Table S15** Expression profiles of genes located in the QTL regions in different tissues.
**Table S16** Preferential expression of genes in different tissues.
**Table S17** Information regarding the reported genes located in the QTL regions.
