# Supplementary material for: Population structure and genetic basis of the agronomic traits of upland cotton in China revealed by a genome‐wide association study using high‐density SNPs
Source: Plant Biotechnol J. 2017 Apr 12;15(11):1374–86. doi: 10.1111/pbi.12722 (PMC5633765; doi:10.1111/pbi.12722)
Supplement: Supplementary file 1 — Figure S1 Curve chart of linkage disequilibrium (LD) decay for each of the 26 chromosomes. (a) LD decay for 13 At‐genome chromosomes: Chr01‐Chr13. (b) LD decay for 13 Dt‐genome chromosomes: Chr14‐Chr26. Figure S2 Distribution of the three subpopulations in 7 region‐clusters and 4 breeding period‐clusters. (a) Percentages stacked column chart for 7 regions: YRR, YtRR, NIR, NSEMR, SCR, USA and SU. (b) Percentages stacked column chart for four breeding periods: S1, S2, S3 and S4. Figure S3 Round tree for the 503 accessions. The outer ring represents three subpopulations; the inner ring represents 7 regions. Figure S4 Principal component analysis of 503 accessions based on phenotype. (a) Scatter plot for three subpopulations: Group 1, Group 2 and Group 3. (b) Scatter plot for 7 regions: YRR, YtRR, NIR, NSEMR, SCR, USA and SU. (c) Scatter plot for four breeding periods: S1, S2, S3 and S4. Figure S5 Summary of GWAS results for flowering period (FP). (a) Phenotype histogram for FP. (b) Q‐Q plots for FP using GLM, GLM (Q), GLM (PCA), MLM (K), MLM (PCA+K), and MLM (Q+K). (c) Manhattan plot for FP GWAS results. The threshold value was set at p < 10−4.078. Figure S6 Summary of GWAS results for the entire growth period (WGP). (a) Phenotype histogram for WGP. (b) Q‐Q plots for WGP using GLM, GLM (Q), GLM (PCA), MLM (K), MLM (PCA+K), and MLM (Q+K). (c) Manhattan plot for WGP GWAS results. The threshold value was set at p < 10−4.078. Figure S7 Summary of GWAS results for first fruit spur height (FFSH). (a) Phenotype histogram for FFSH. (b) Q‐Q plots for FFSH using GLM, GLM (Q), GLM (PCA), MLM (K), MLM (PCA+K), and MLM (Q+K). (c) Manhattan plot for FFSH GWAS results. The threshold value was set at p < 10−4.078. Figure S8 Summary of GWAS results for fruit spur branch number (FSBN). (a) Phenotype histogram for FFBN. (b) Q‐Q plots for FSBN using GLM, GLM (Q), GLM (PCA), MLM (K), MLM (PCA+K), and MLM (Q+K). (c) Manhattan plot for FSBN GWAS results. The threshold value was set at p < 10 [file PBI-15-1374-s001.pdf]

Supplementary Figure 1-23

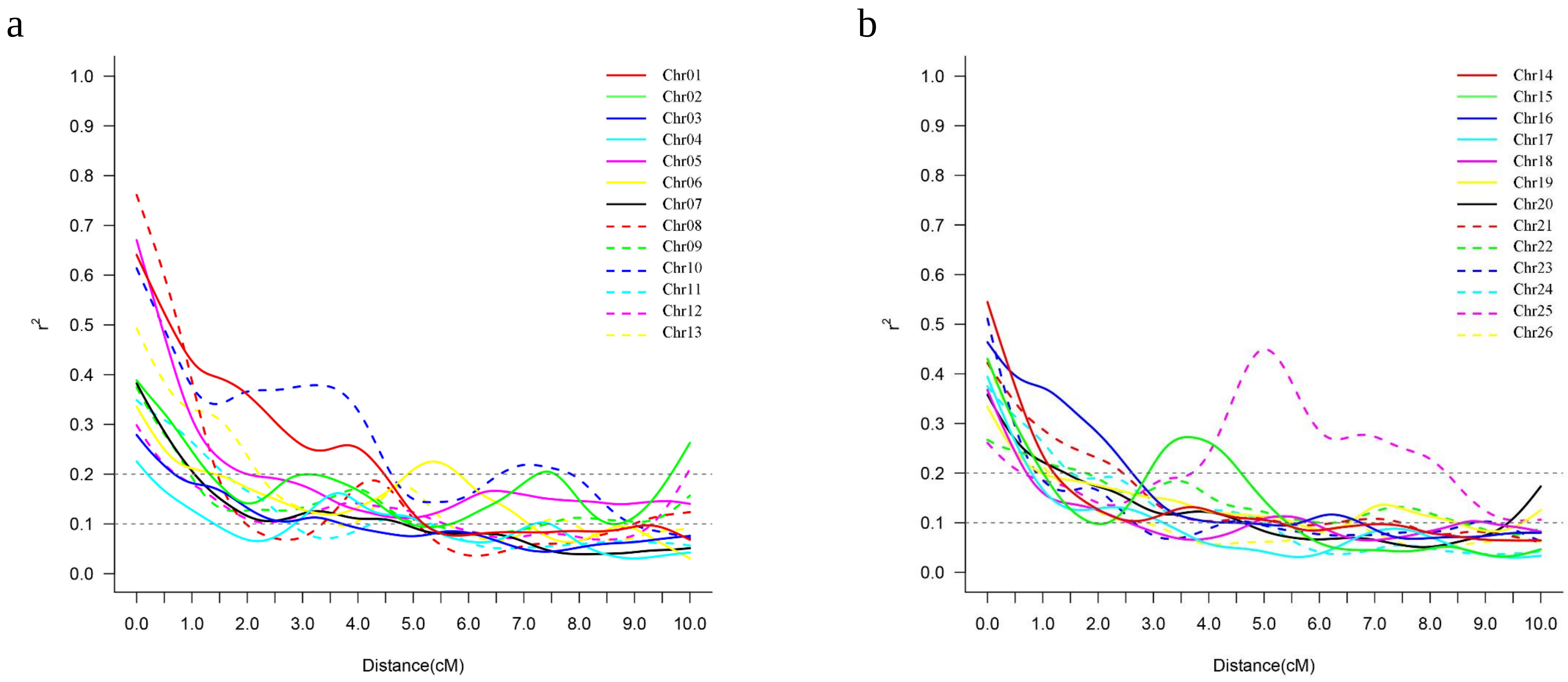

Figure S1. Curve chart of linkage disequilibrium (LD) decay for each of the26 chromosomes. (a) LD decay for 13 A-genome chromosomes :Chr01-Chr13. (b) LD decay for 13 D-genome chromosomes :Chr14-Chr26.

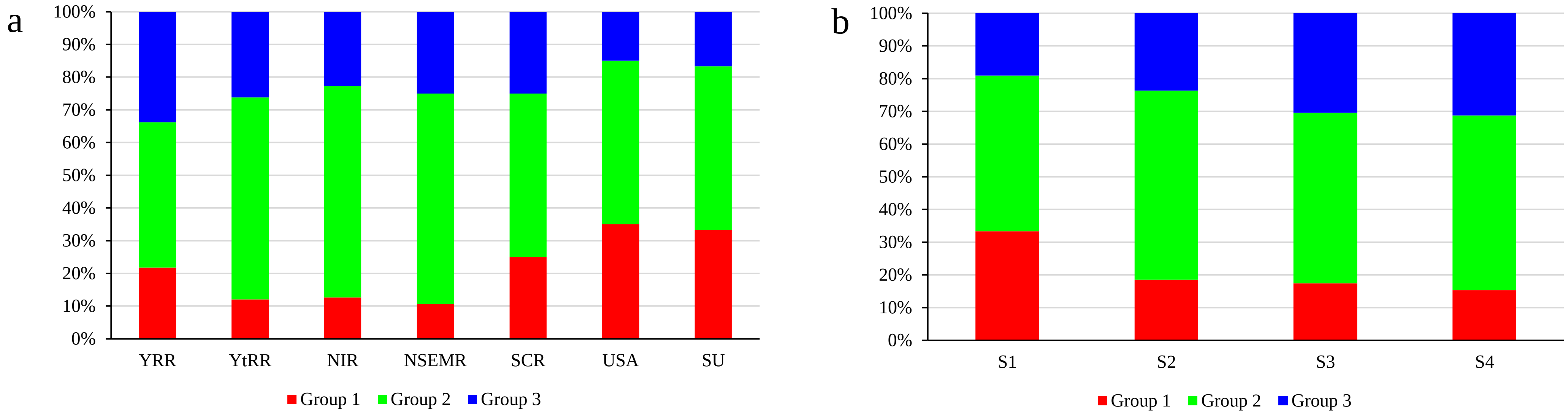

Figure S2. Distribution of the three subpopulations in 7 region-clusters and 4 breeding period-clusters. (a) Percentages stacked column chart for 7 regions: YRR, YtRR, NIR, NSEMR, SCR, USA and SU. (b) Percentages stacked column chart for four breeding periods: S1, S2, S3 and S4.

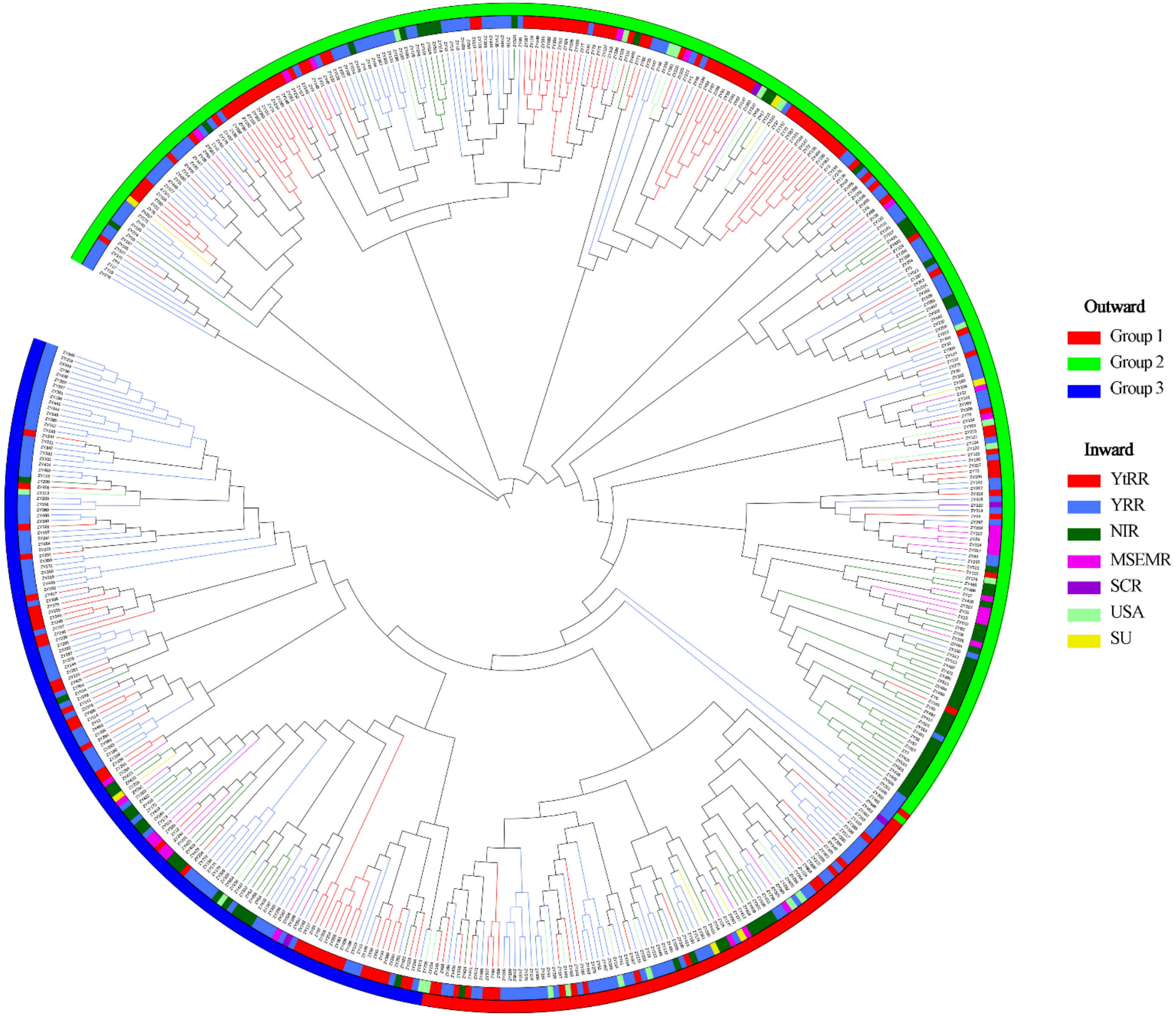

Figure S3. Round tree for 503 accessions. The outer ring represents three subpopulations; inner ring represents 7 regions..

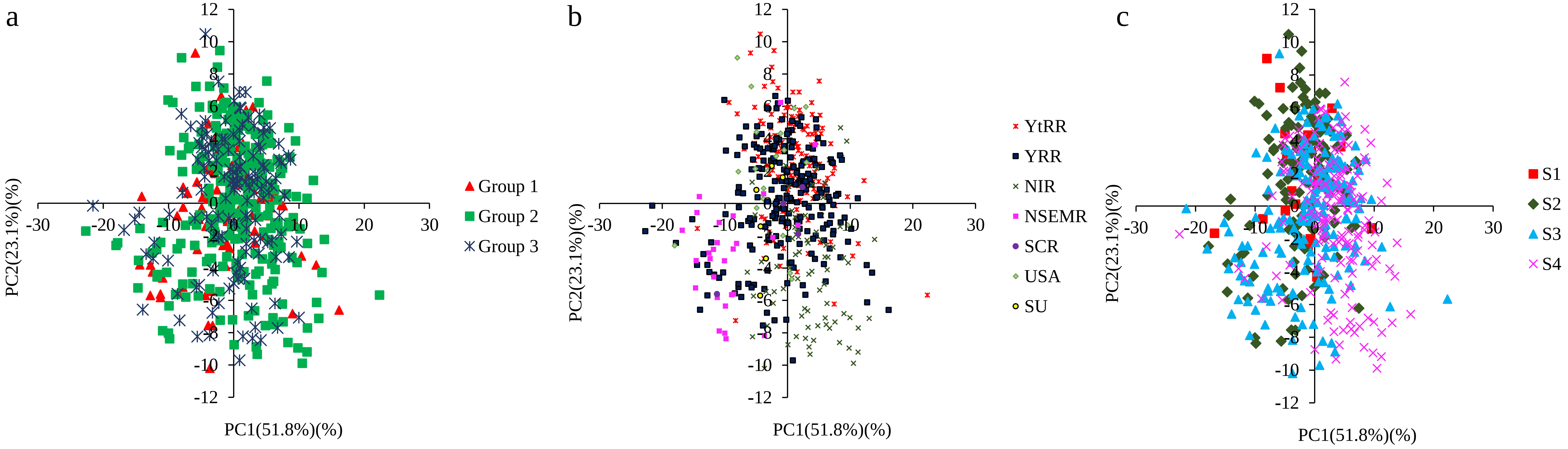

Figure S4. Principal component analysis of 503 accessions based on phenotype. (a) Scatter plot for three subpopulations: Group 1, Group 2, Group 3. (b) Scatter plot for 7 regions: YRR, YtRR, NIR, NSEMR, SCR, USA and SU. (c) Scatter plot for four breeding periods: S1, S2, S3 and S4.

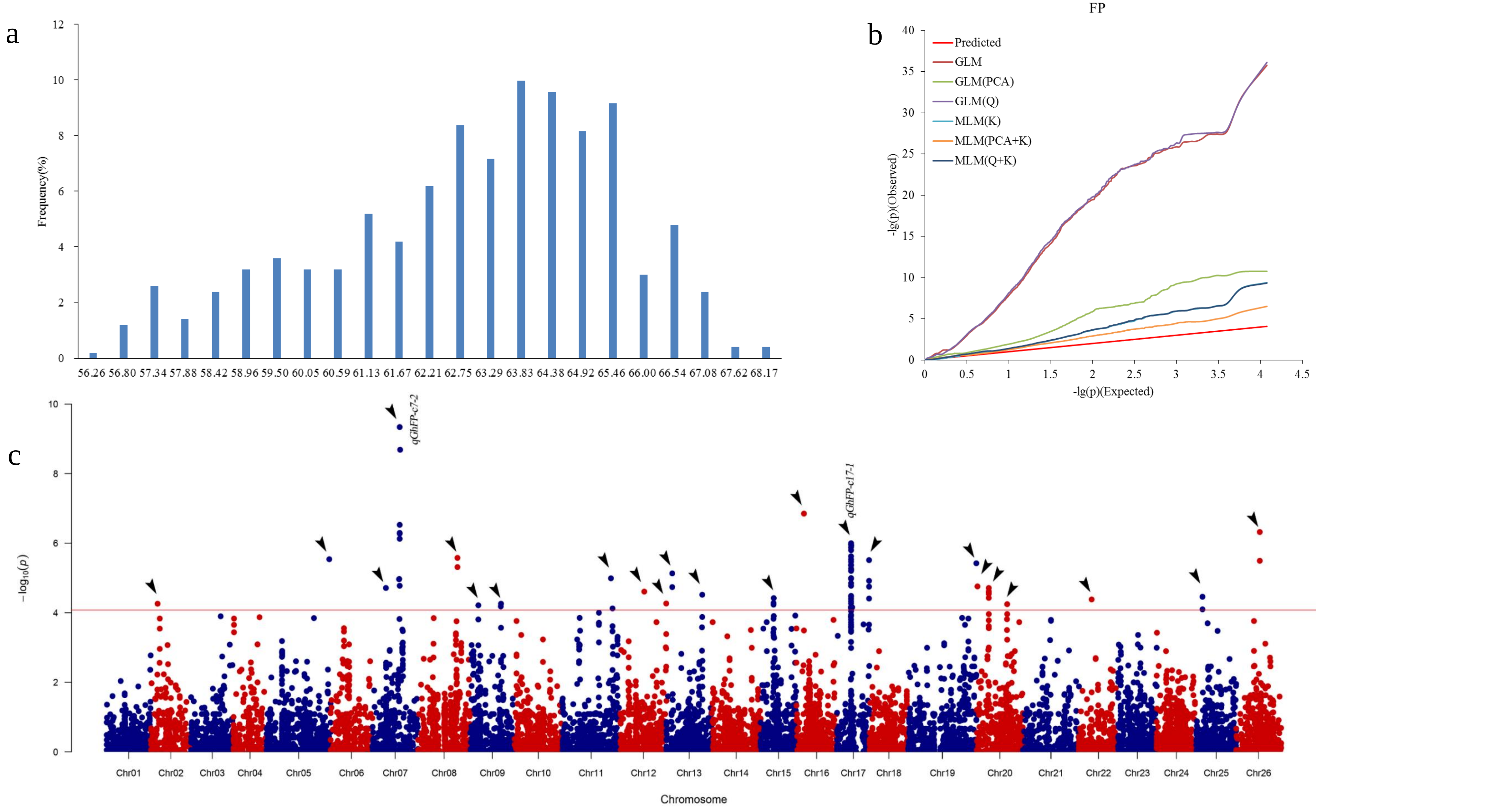

Figure S5. Summary of GWAS results for flowering period (FP). (a) Phenotype histogram for FP. (b) Q-Q plots for FP using GLM, GLM (Q), GLM (PCA), MLM (K), MLM (PCA+K), and MLM (Q+K). (c) Manhattan plot for FP GWAS results. The threshold value was set at $p < 10^{-4.078}$.

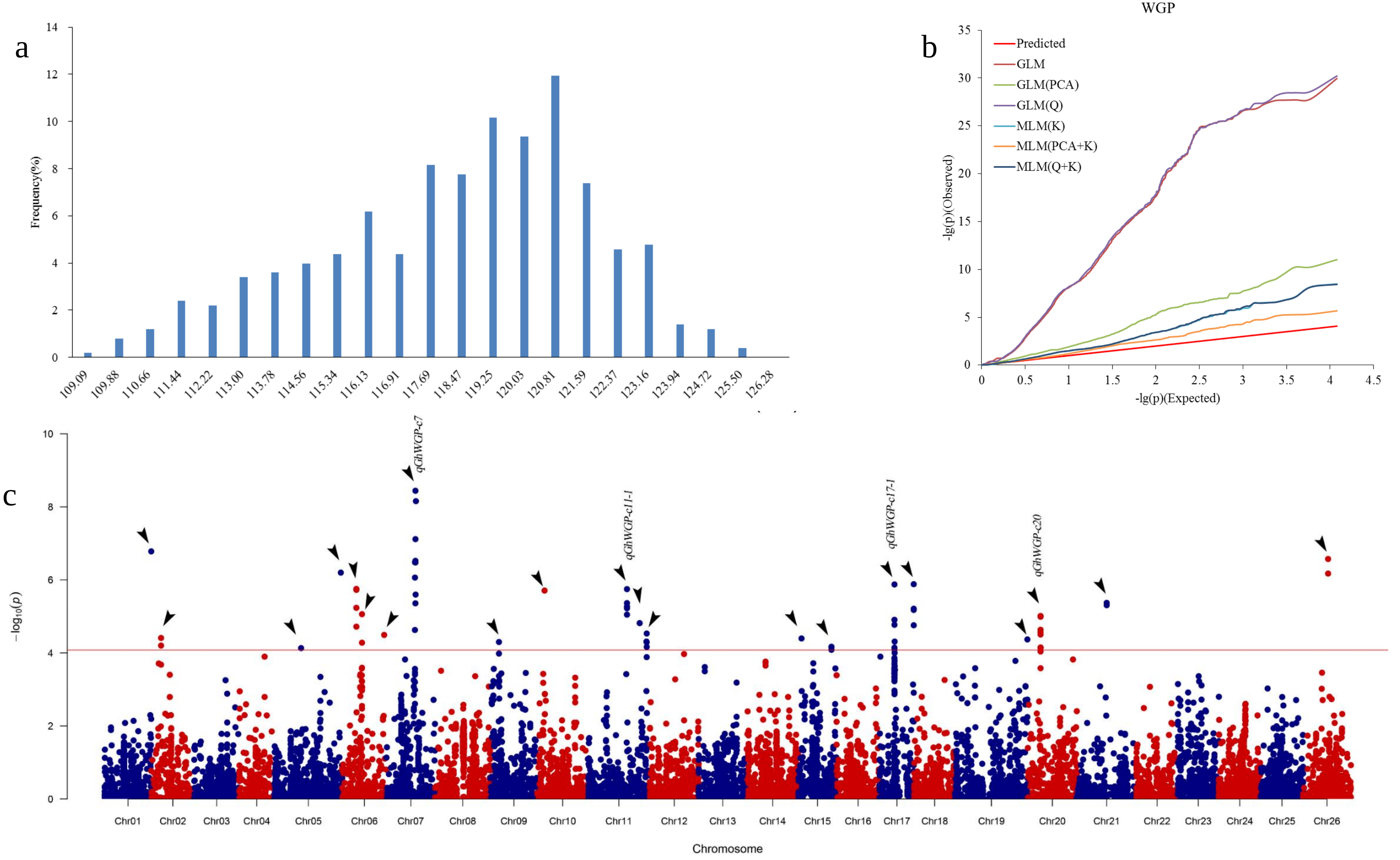

Figure S6. Summary of GWAS results for whole growth period (WGP). (a) Phenotype histogram for WGP. (b) Q-Q plots for WGP using GLM, GLM (Q), GLM (PCA), MLM (K), MLM (PCA+K), and MLM (Q+K). (c) Manhattan plot for WGP GWAS results. The threshold value was set at $p < 10^{-4.078}$.

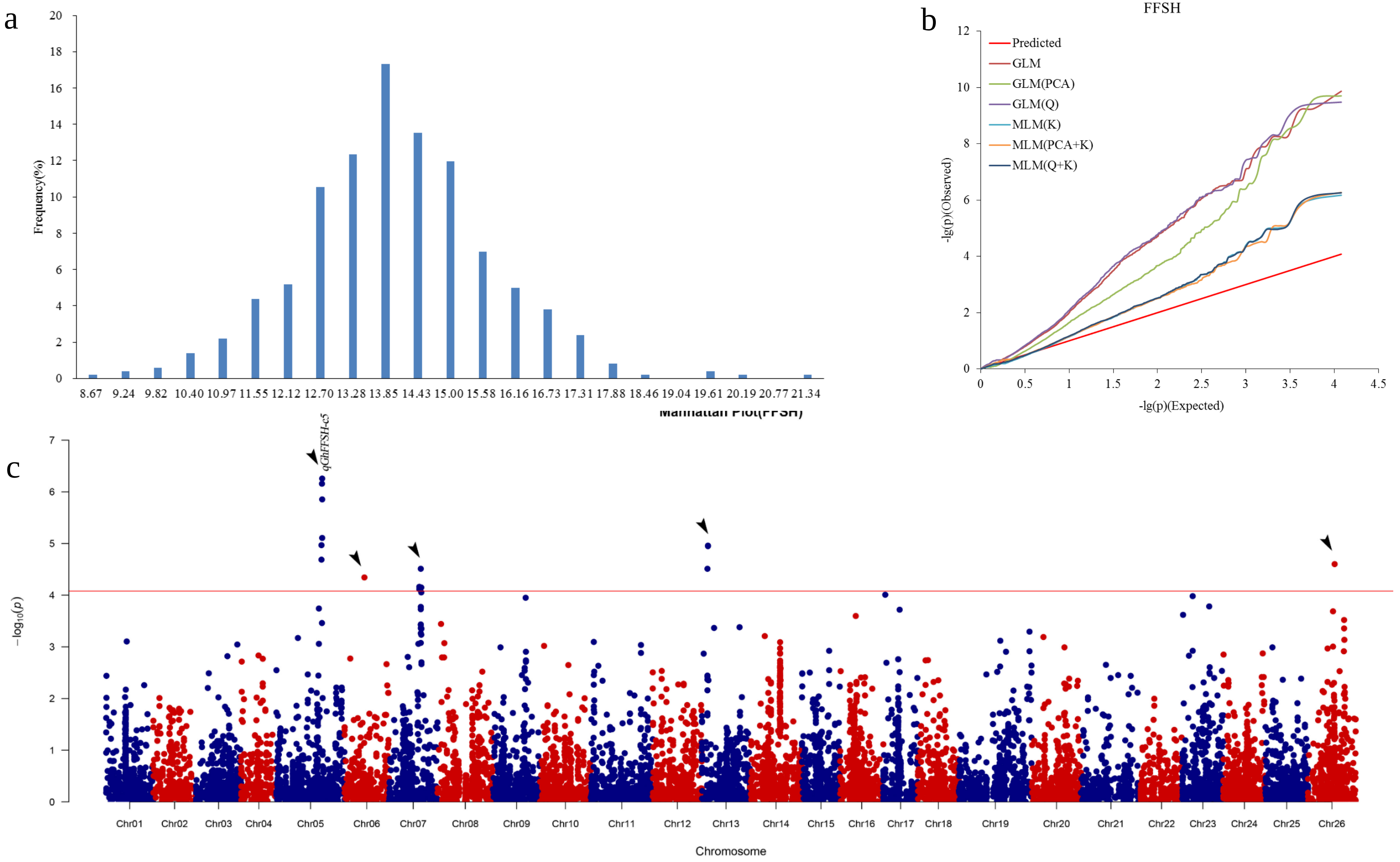

Figure S7. Summary of GWAS results for first fruit spur height (FFSH). (a) Phenotype histogram for FFSH. (b) Q-Q plots for FFSH using GLM, GLM (Q), GLM (PCA), MLM (K), MLM (PCA+K), and MLM (Q+K). (c) Manhattan plot for FFSH GWAS results. The threshold value was set at $p < 10^{-4.078}$.

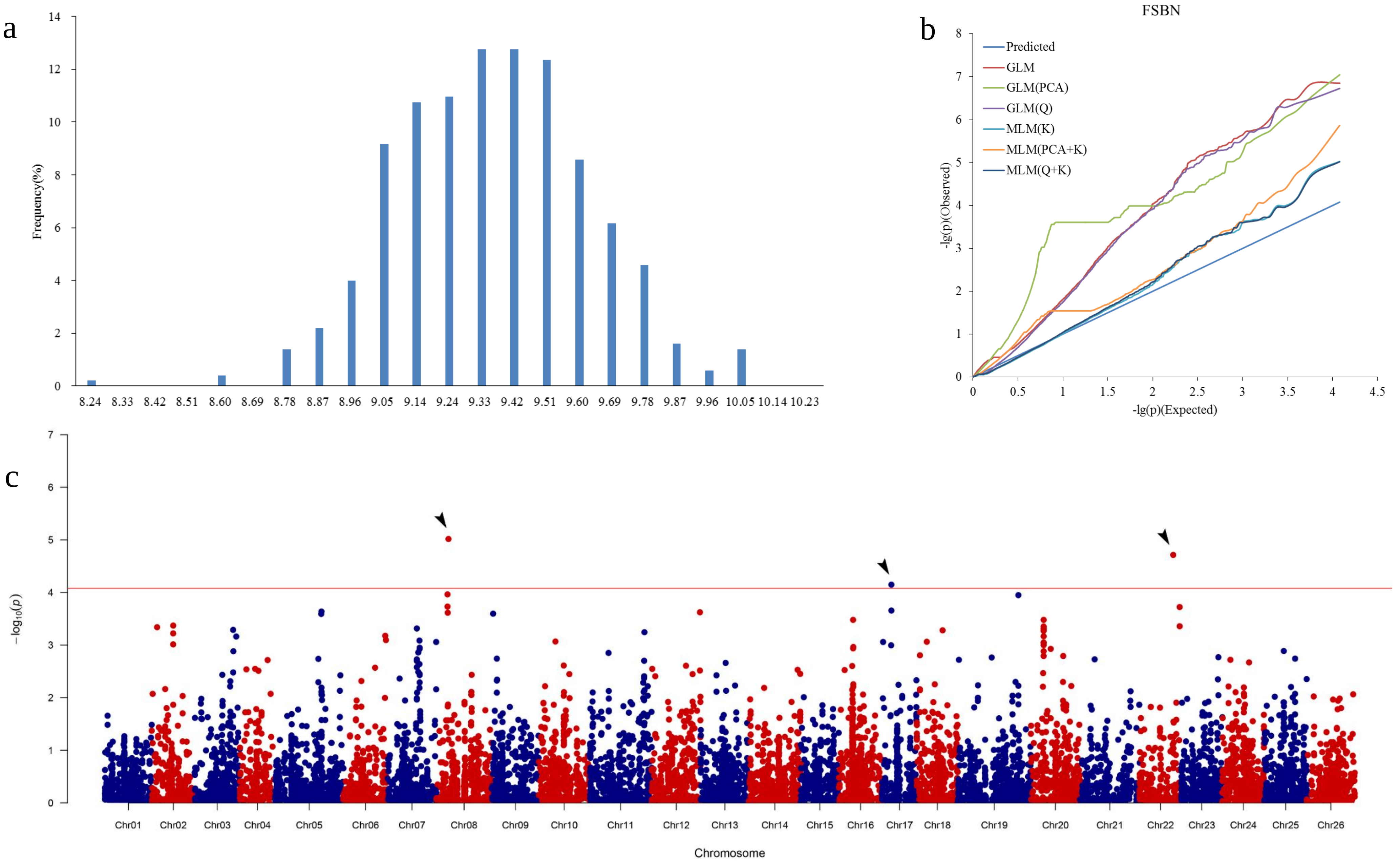

Figure S8. Summary of GWAS results for fruit spur branch number (FSBN). (a) Phenotype histogram for FFBN. (b) Q-Q plots for FSBN using GLM, GLM (Q), GLM (PCA), MLM (K), MLM (PCA+K), and MLM (Q+K). (c) Manhattan plot for FSBN GWAS results. The threshold value was set at $p < 10^{-4.078}$.

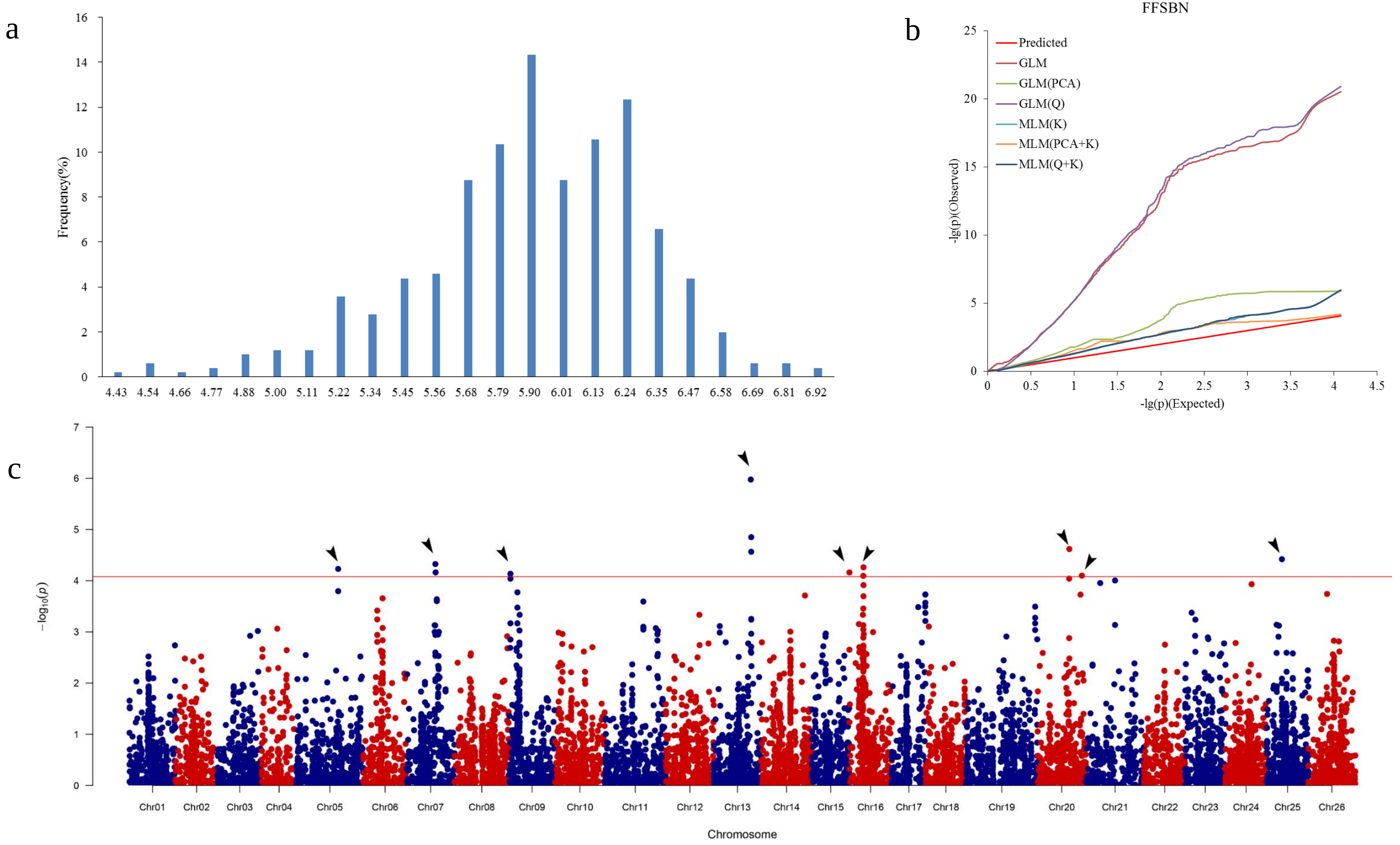

Figure S9. Summary of GWAS results first fruit spur branch number (FFSBN). (a) Phenotype histogram for FFSBN. (b) Q-Q plots for FFSBN using GLM, GLM (Q), GLM (PCA), MLM (K), MLM (PCA+K), and MLM (Q+K). (c) Manhattan plot for FFSBN GWAS results. The threshold value was set at $p < 10^{-4.078}$.

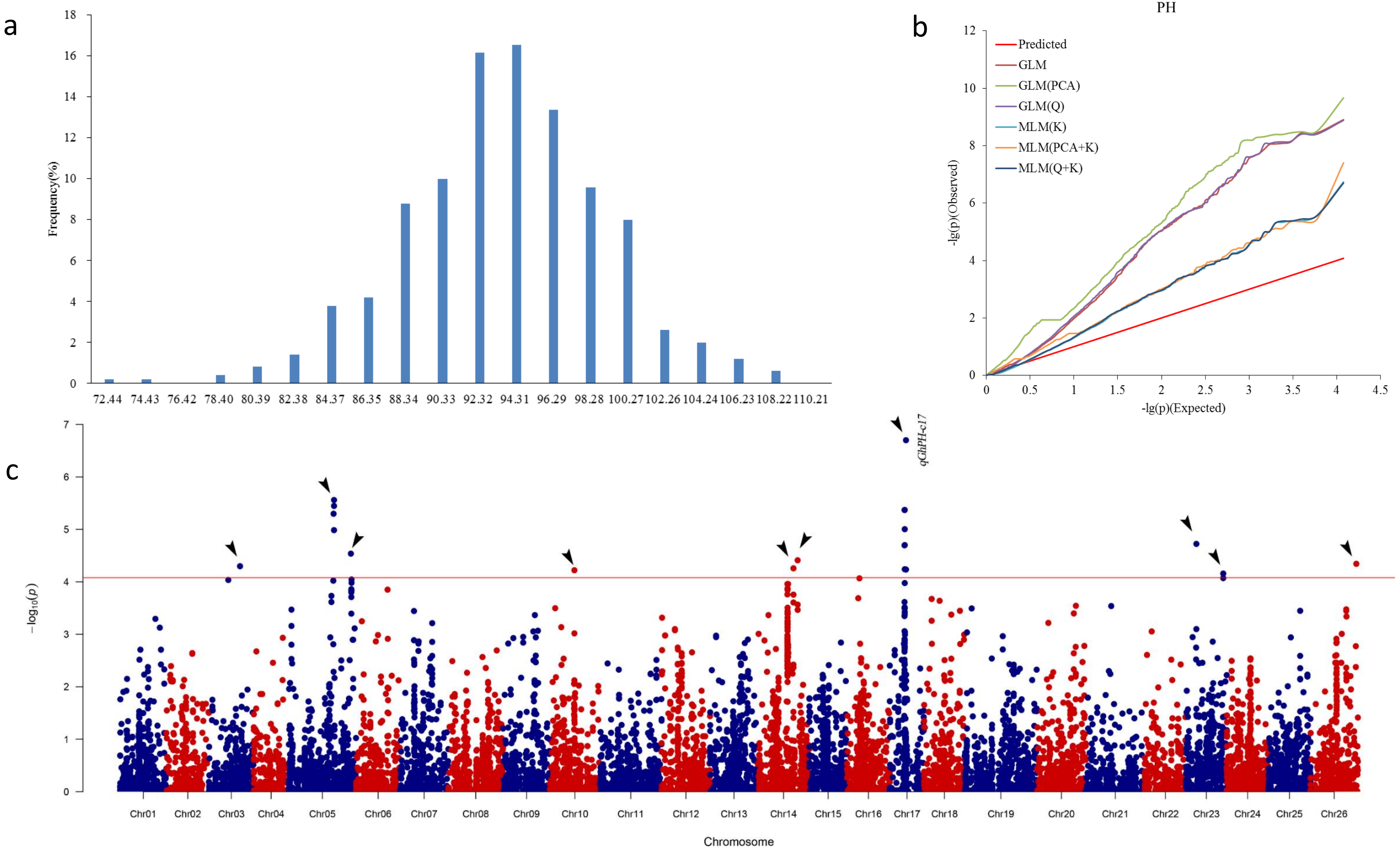

Figure S10. Summary of GWAS results for plant height (PH). (a) Phenotype histogram for PH. (b) Q-Q plots for PH using GLM, GLM (Q), GLM (PCA), MLM (K), MLM (PCA+K), and MLM (Q+K). (c) Manhattan plot for PH GWAS results. The threshold value was set at $p < 10^{-4.078}$.

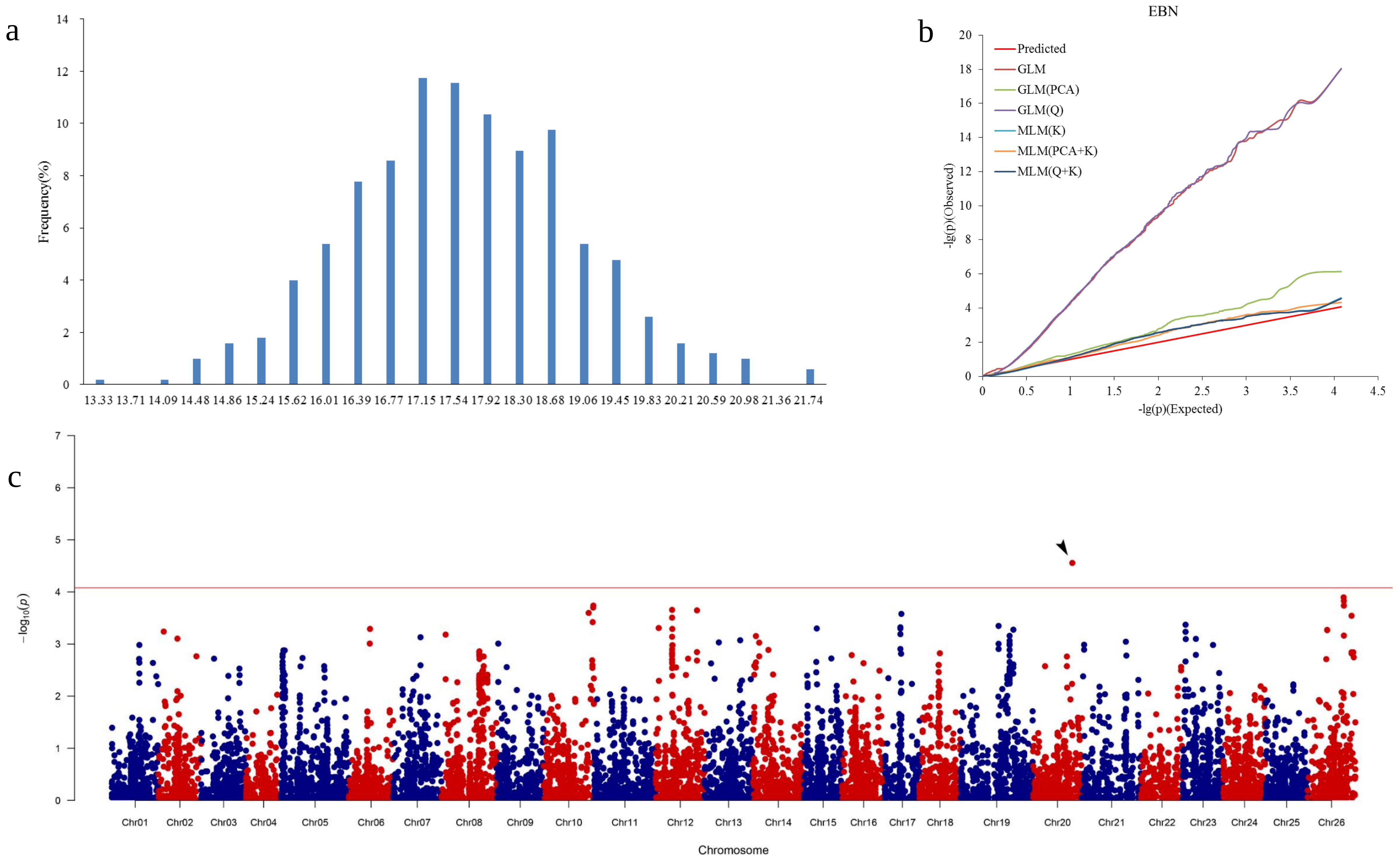

Figure S11. Summary of GWAS results for effective boll number (EBN). (a) Phenotype histogram for EBN. (b) Q-Q plots for EBN using GLM, GLM (Q), GLM (PCA), MLM (K), MLM (PCA+K), and MLM (Q+K). (c) Manhattan plot for EBN GWAS results. The threshold value was set at $p < 10^{-4.078}$.

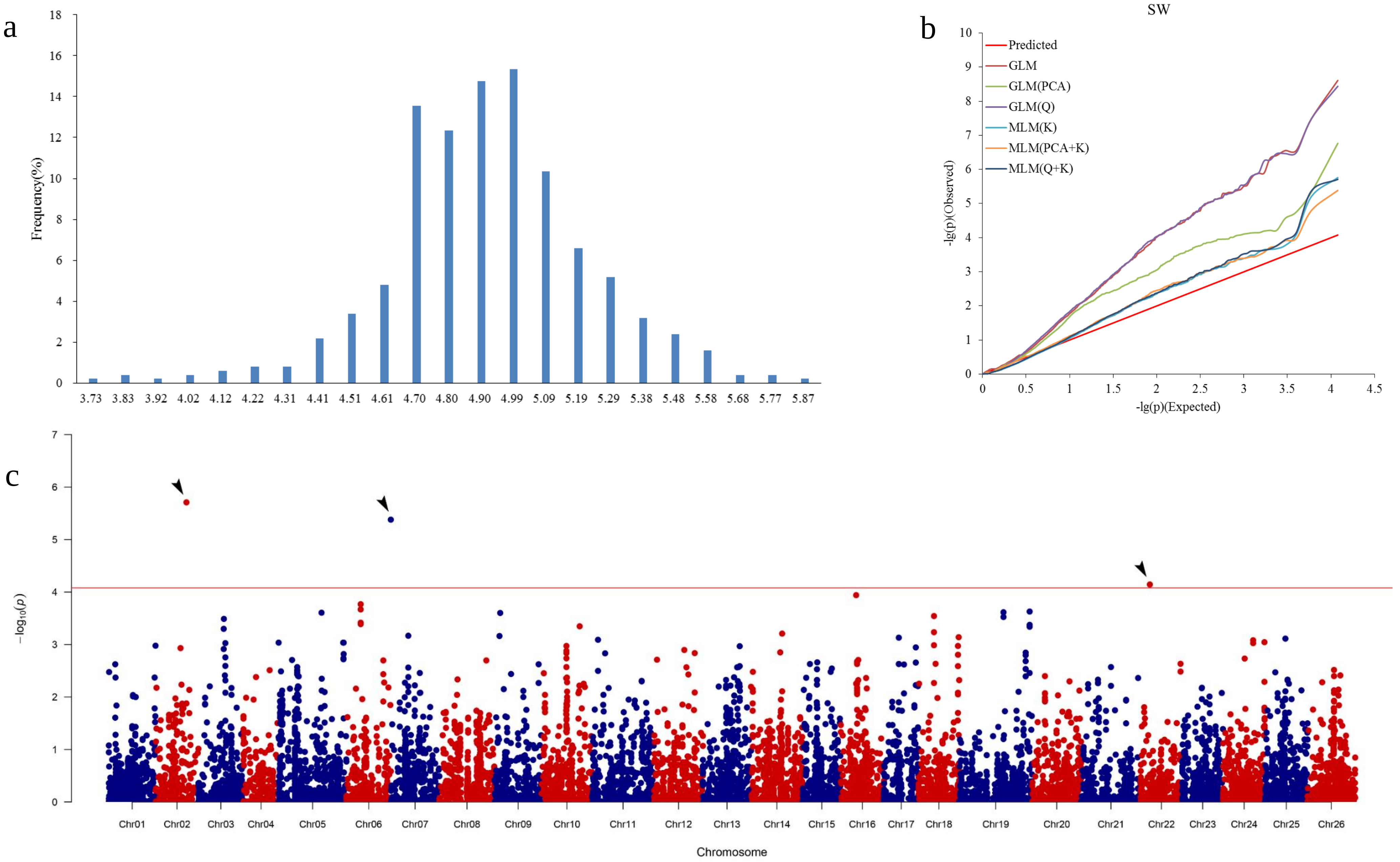

Figure S12. Summary of GWAS results for seed weight (SW). (a) Phenotype histogram for SW. (b) Q-Q plots for SW using GLM, GLM (Q), GLM (PCA), MLM (K), MLM (PCA+K), and MLM (Q+K). (c) Manhattan plot for SW GWAS results. The threshold value was set at $p < 10^{-4.078}$.

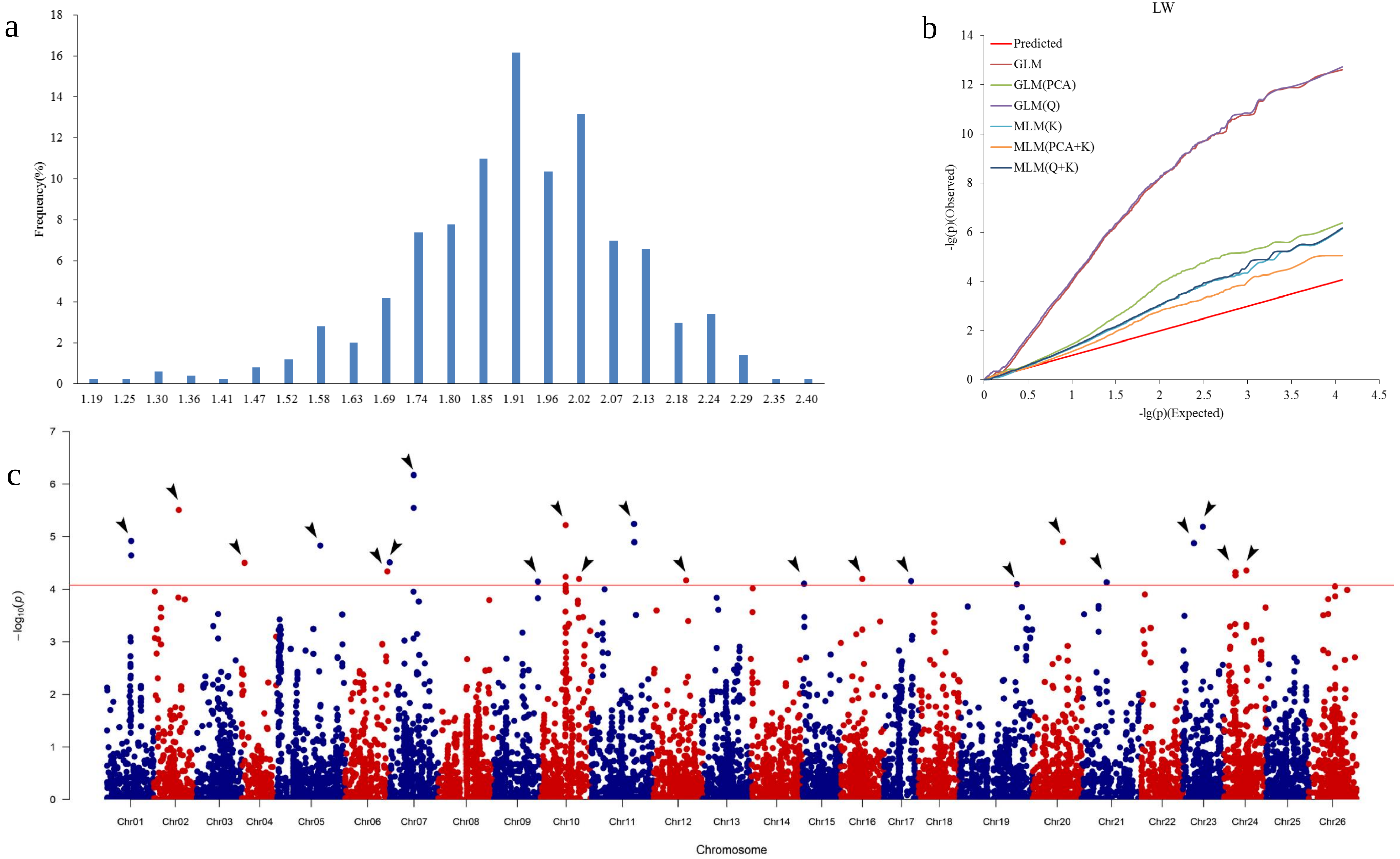

Figure S13. Summary of GWAS results for lint weight (LW). (a) Phenotype histogram for LW. (b) Q-Q plots for LW using GLM, GLM (Q), GLM (PCA), MLM (K), MLM (PCA+K), and MLM (Q+K). (c) Manhattan plot for LW GWAS results. The threshold value was set at $p < 10^{-4.078}$.

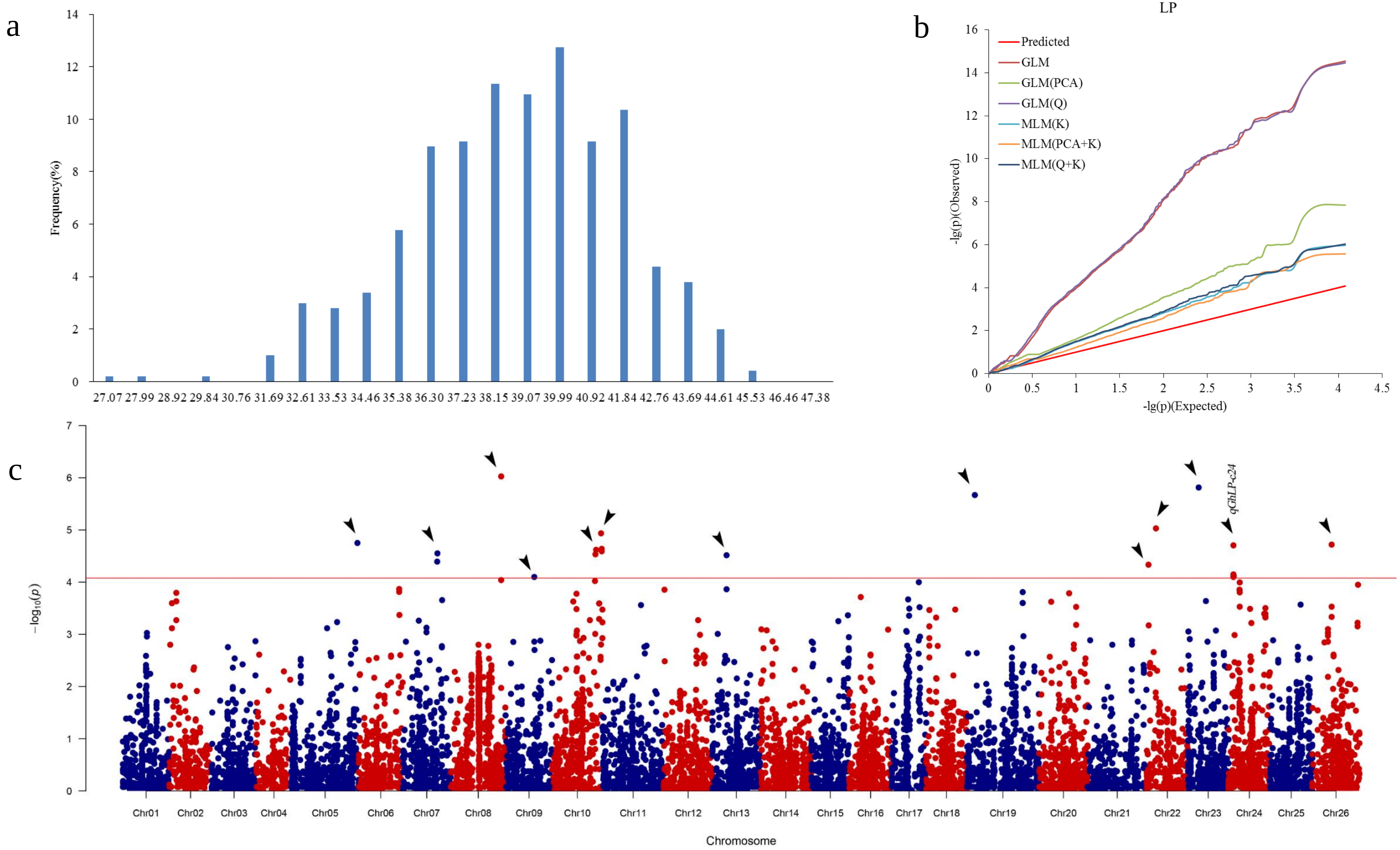

Figure S14. Summary of GWAS results for lint percentage (LP). (a) Phenotype histogram for LP. (b) Q-Q plots for LP using GLM, GLM (Q), GLM (PCA), MLM (K), MLM (PCA+K), and MLM (Q+K). (c) Manhattan plot for LP GWAS results. The threshold value was set at $p < 10^{-4.078}$.

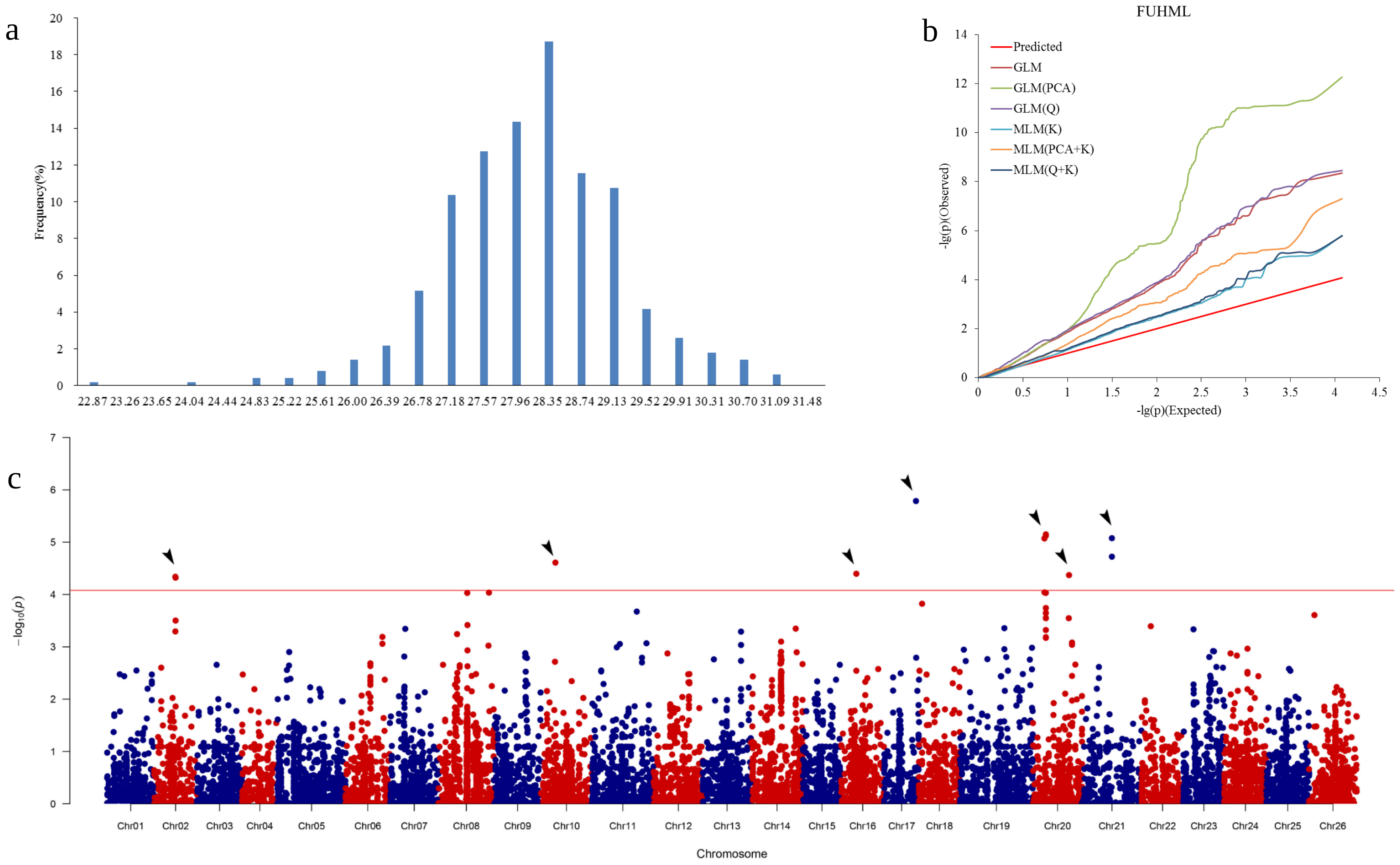

Figure S15. Summary of GWAS results for fibre upper half mean length (FUHML). (a) Phenotype histogram for FUHML. (b) Q-Q plots for FUHML using GLM, GLM (Q), GLM (PCA), MLM (K), MLM (PCA+K), and MLM (Q+K). (c) Manhattan plot for FUHML GWAS results. The threshold value was set at $p < 10^{-4.078}$.

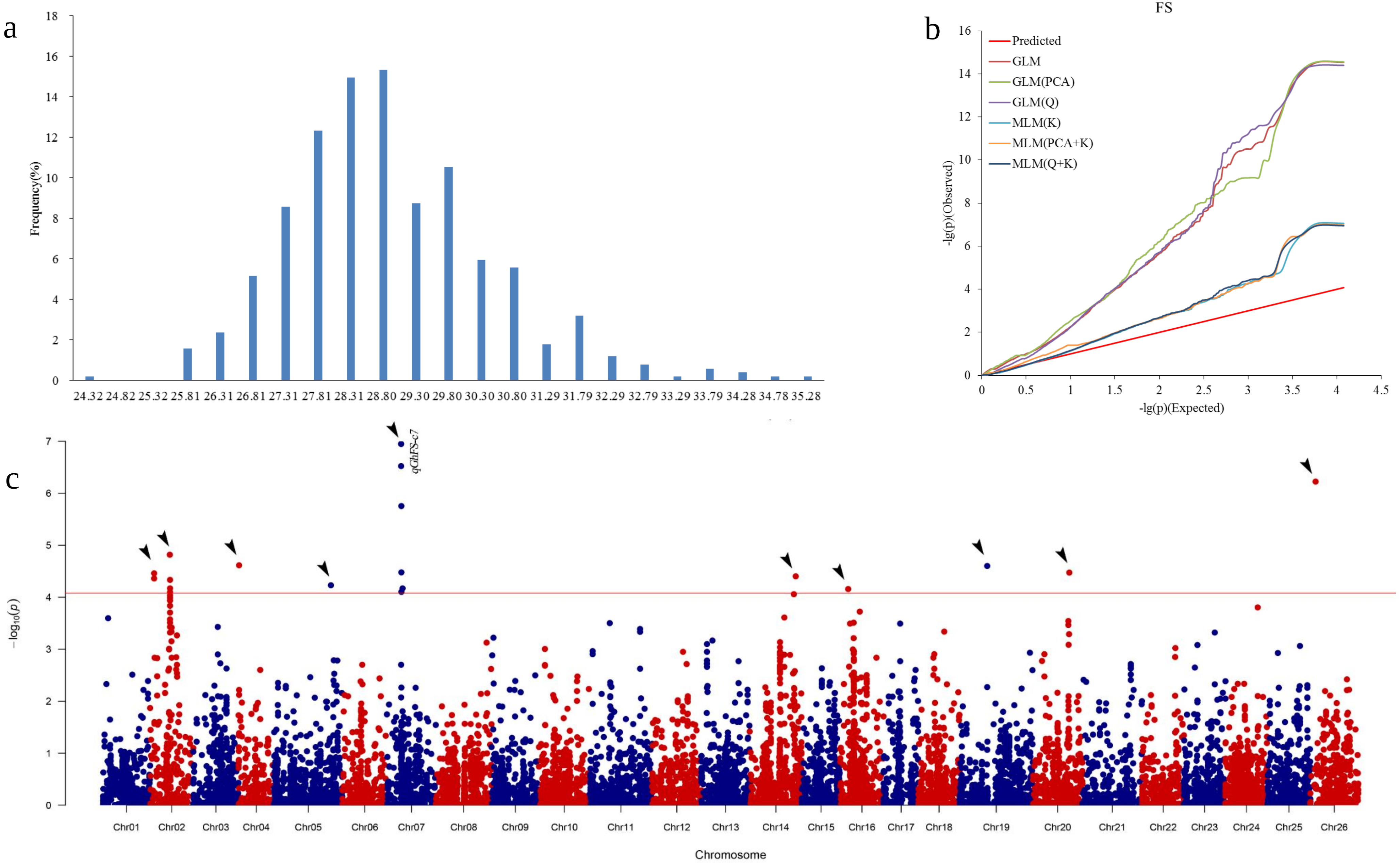

Figure S16. Summary of GWAS results for fibre strength (FS). (a) Phenotype histogram for FS. (b) Q-Q plots for FS using GLM, GLM (Q), GLM (PCA), MLM (K), MLM (PCA+K), and MLM (Q+K). (c) Manhattan plot for FS GWAS results. The threshold value was set at $p < 10^{-4.078}$.

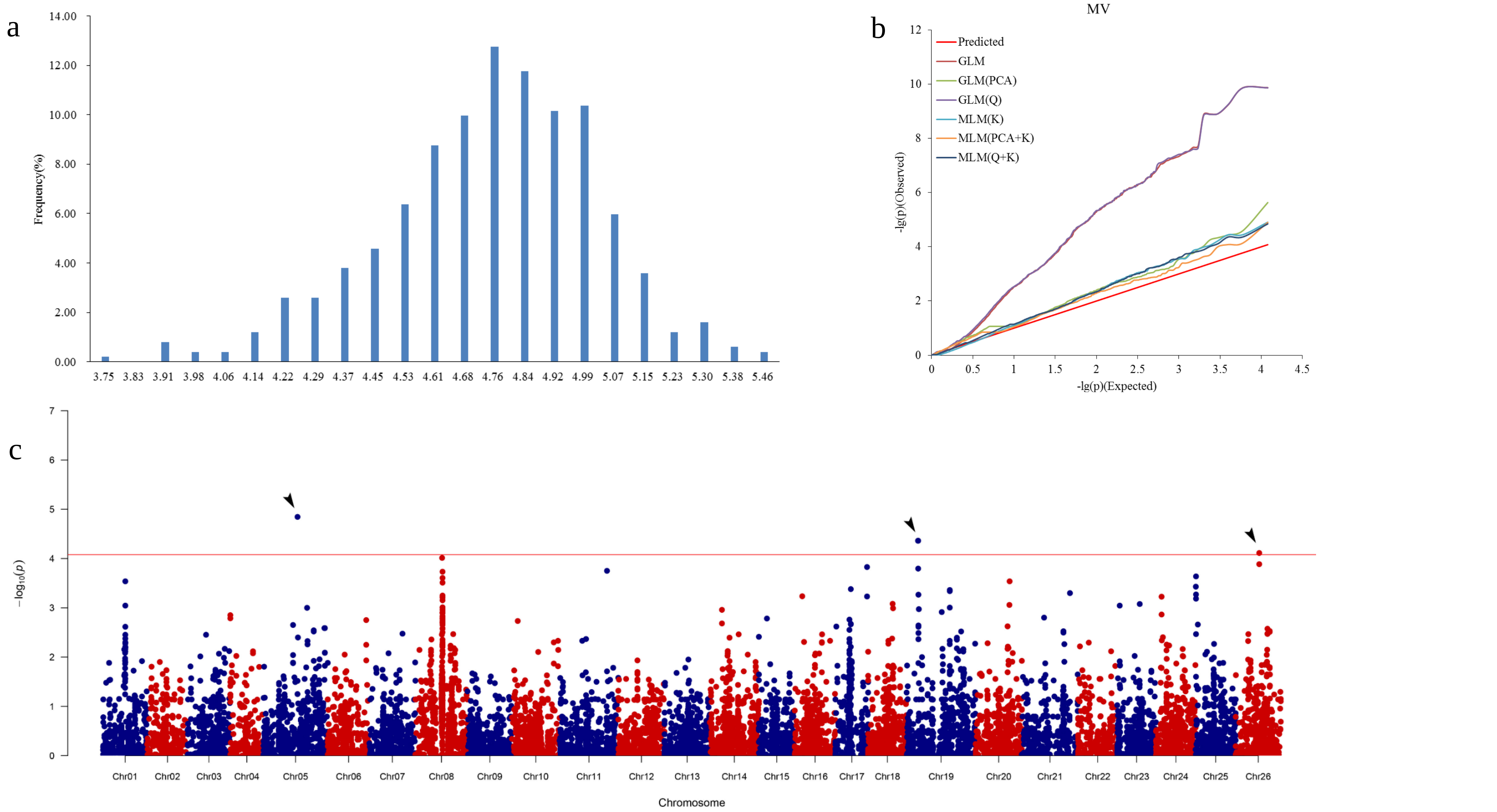

Figure S17. Summary of GWAS results for micronaire value (MV). (a) Phenotype histogram for MV. (b) Q-Q plots for MV using GLM, GLM (Q), GLM (PCA), MLM (K), MLM (PCA+K), and MLM (Q+K). (c) Manhattan plot for MV GWAS results. The threshold value was set at $p < 10^{-4.078}$.

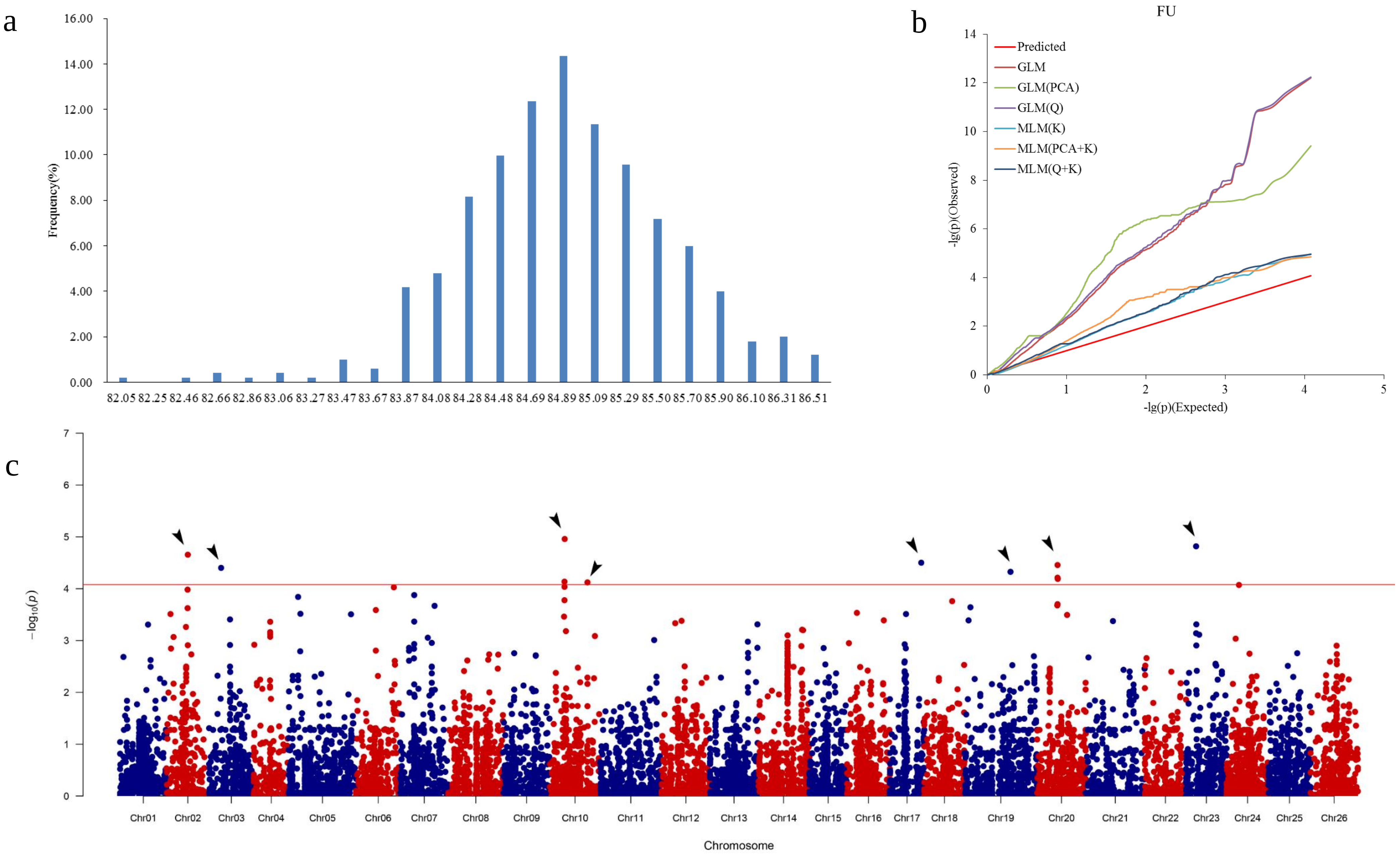

Figure S18. Summary of GWAS results for fibre uniformity (FU). (a) Phenotype histogram for FU. (b) Q-Q plots for FU using GLM, GLM (Q), GLM (PCA), MLM (K), MLM (PCA+K), and MLM (Q+K). (c) Manhattan plot for FU GWAS results. The threshold value was set at $p < 10^{-4.078}$.

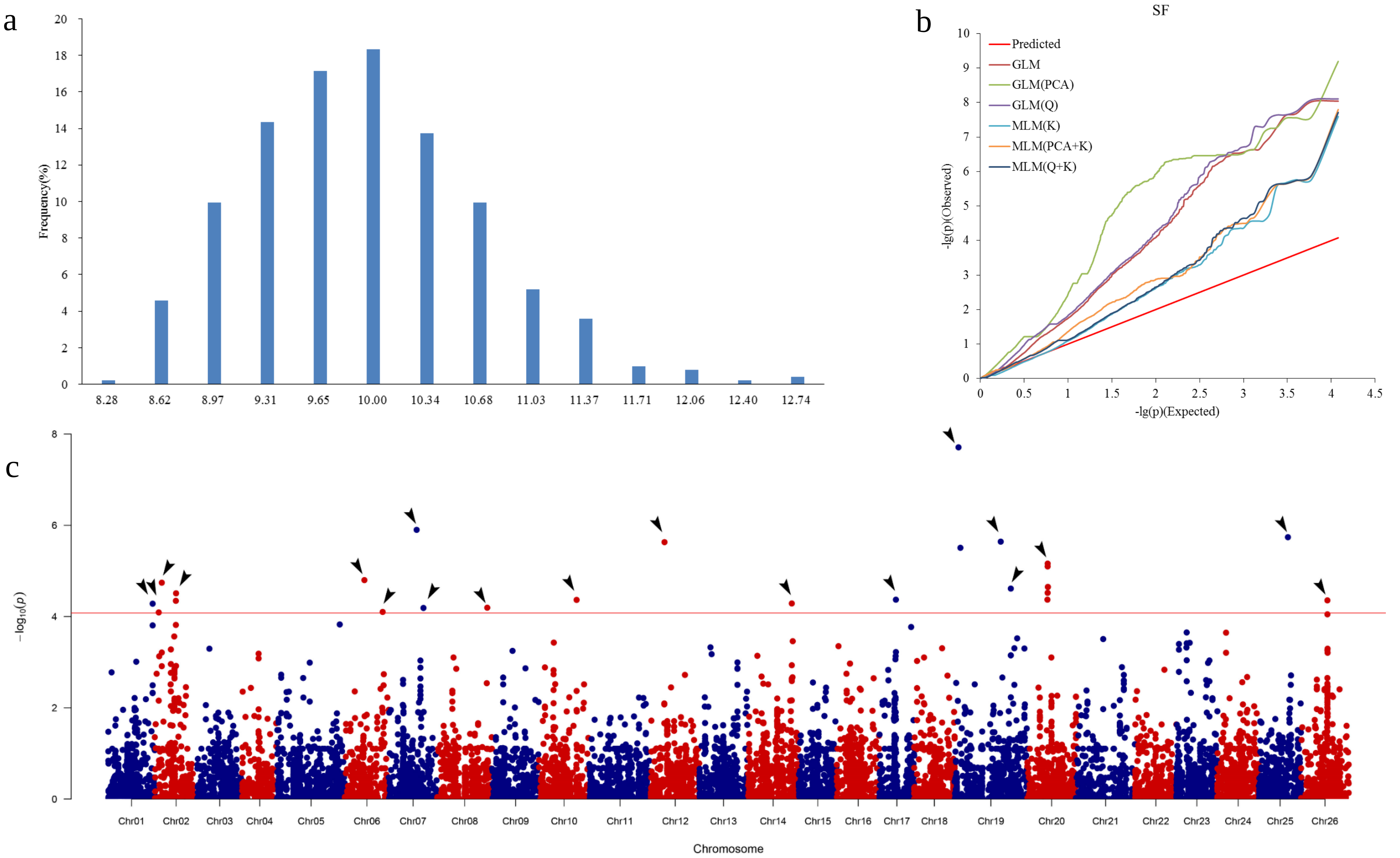

Figure S19. Summary of GWAS results for short fibre percentage (SF). (a) Phenotype histogram for SF. (b) Q-Q plots for SF using GLM, GLM (Q), GLM (PCA), MLM (K), MLM (PCA+K), and MLM (Q+K). (c) Manhattan plot for SF GWAS results. The threshold value was set at $p < 10^{-4.078}$.

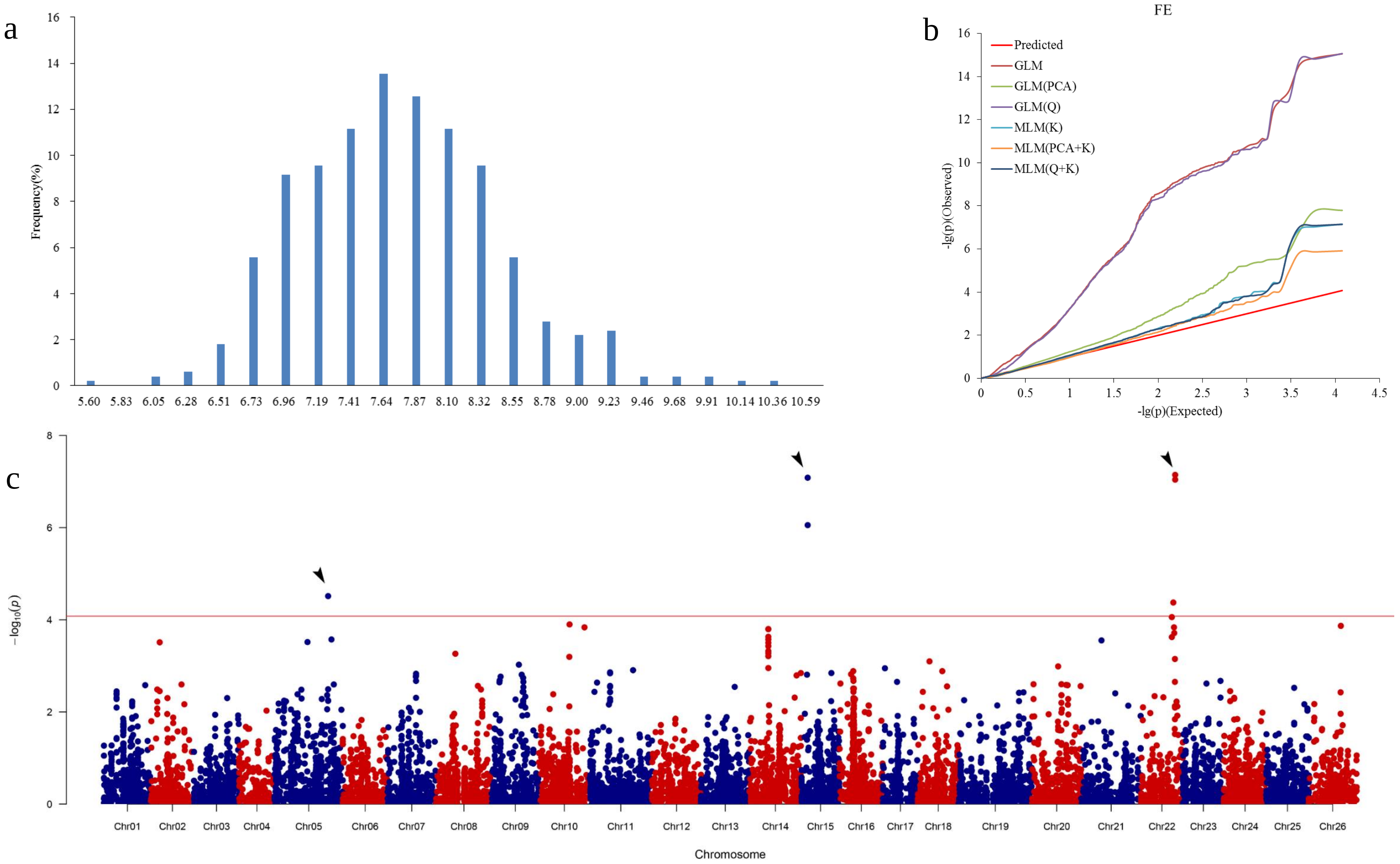

Figure S20. Summary of GWAS results for fibre elongation (FE). (a) Phenotype histogram for FE. (b) Q-Q plots for FE using GLM, GLM (Q), GLM (PCA), MLM (K), MLM (PCA+K), and MLM (Q+K). (c) Manhattan plot for FE GWAS results. The threshold value was set at $p < 10^{-4.078}$.

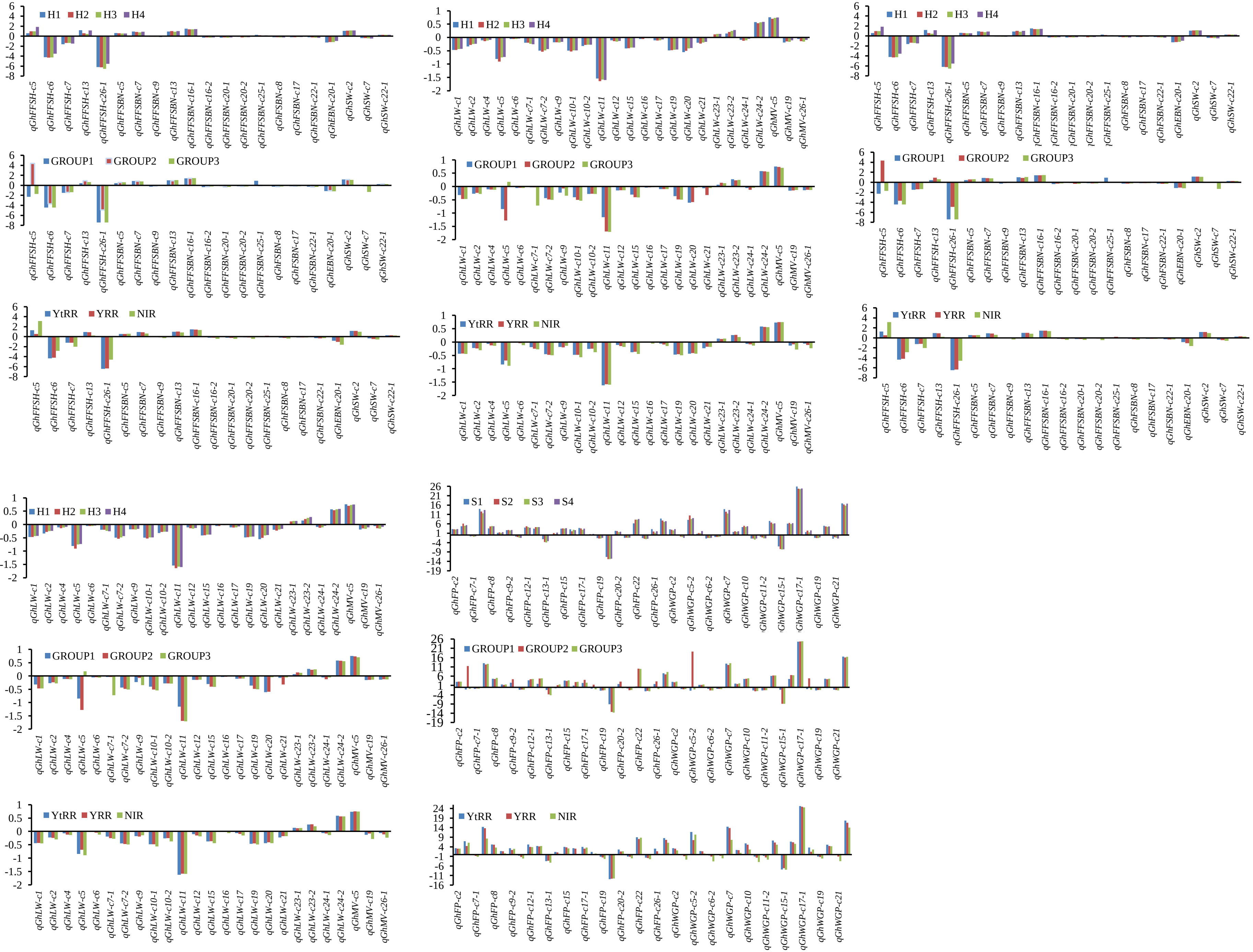

Figure S21. Association loci effects in different clusters.

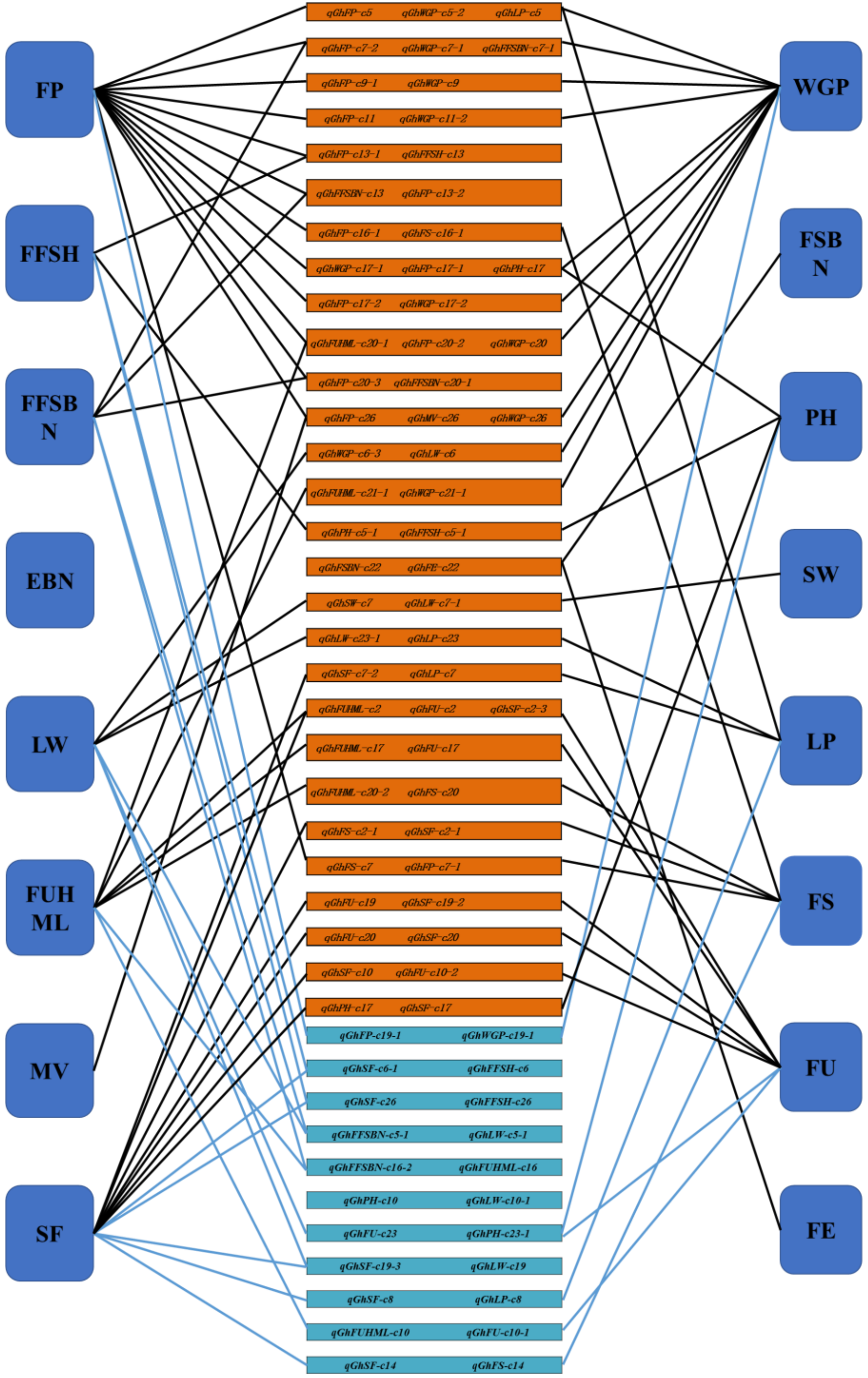

Figure S22. Pleiotropic effects of GWAS QTLs. Orange represents overlapped QTLs for different traits, and blue represents the closely linked QTLs or different traits..

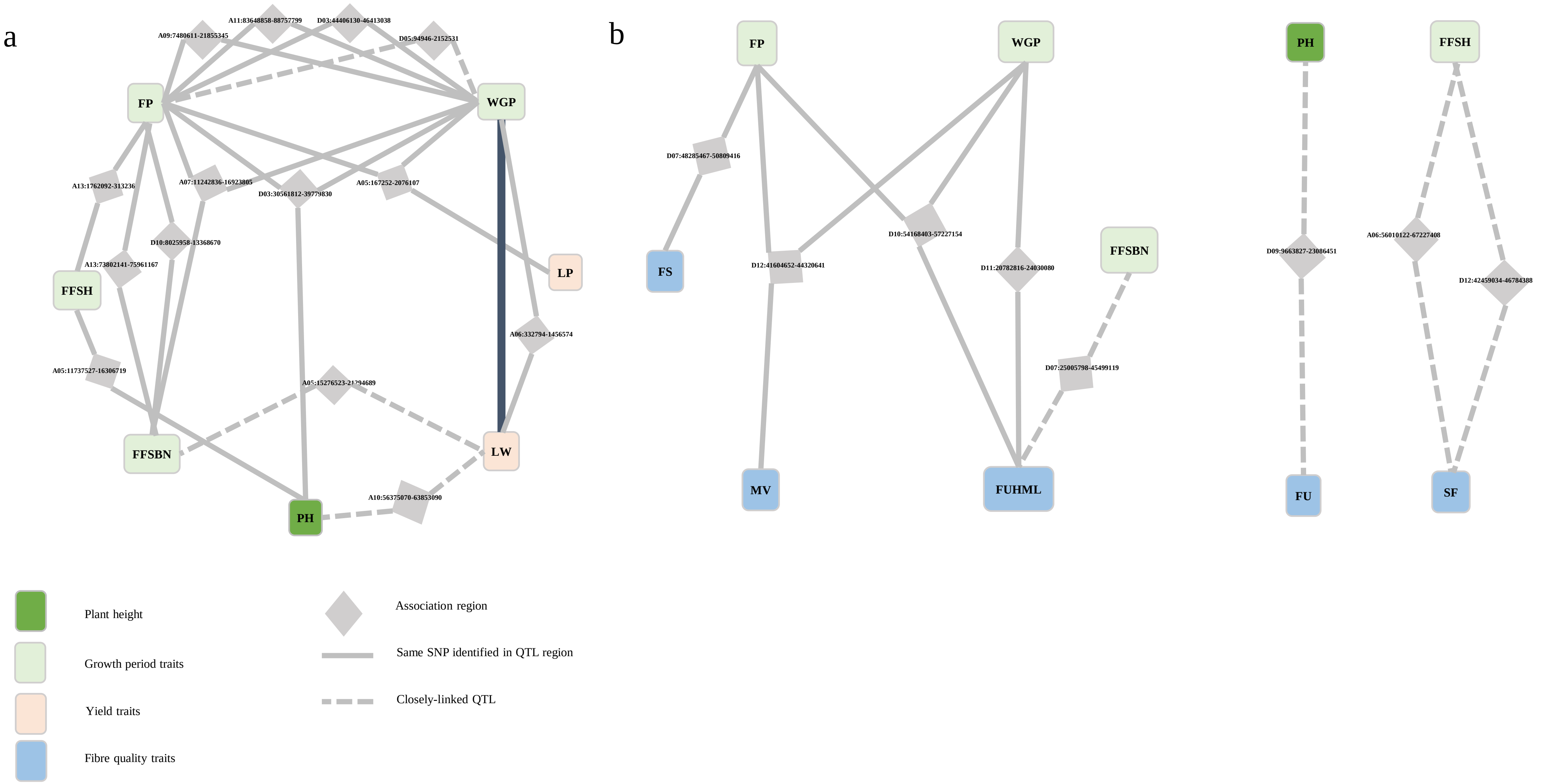

Figure S23. Network dissected QTL regions containing associations (a) Growth period traits, plant height (PH) and yield traits. (b) Growth period traits , plant height (PH) and fibre quality traits. FP: flowering period; WGP: whole growth period; FFSH: first fruit spur height; FFSBN: first fruit spur branch number; PH: plant height; LW: lint weight; LP: lint percentage; FUHML: fibre upper half mean length; FS: fibre strength; FU: fibre uniformity; SF: short fibre, FE: fibre elongation.
